# PSAMM: A Portable System for the Analysis of Metabolic Models

**DOI:** 10.1371/journal.pcbi.1004732

**Published:** 2016-02-01

**Authors:** Jon Lund Steffensen, Keith Dufault-Thompson, Ying Zhang

**Affiliations:** Department of Cell and Molecular Biology, College of the Environment and Life Sciences, University of Rhode Island, Kingston, Rhode Island, United States of America; University of Wurzburg, Biocentre, GERMANY

## Abstract

The genome-scale models of metabolic networks have been broadly applied in phenotype prediction, evolutionary reconstruction, community functional analysis, and metabolic engineering. Despite the development of tools that support individual steps along the modeling procedure, it is still difficult to associate mathematical simulation results with the annotation and biological interpretation of metabolic models. In order to solve this problem, here we developed a Portable System for the Analysis of Metabolic Models (PSAMM), a new open-source software package that supports the integration of heterogeneous metadata in model annotations and provides a user-friendly interface for the analysis of metabolic models. PSAMM is independent of paid software environments like MATLAB, and all its dependencies are freely available for academic users. Compared to existing tools, PSAMM significantly reduced the running time of constraint-based analysis and enabled flexible settings of simulation parameters using simple one-line commands. The integration of heterogeneous, model-specific annotation information in PSAMM is achieved with a novel format of YAML-based model representation, which has several advantages, such as providing a modular organization of model components and simulation settings, enabling model version tracking, and permitting the integration of multiple simulation problems. PSAMM also includes a number of quality checking procedures to examine stoichiometric balance and to identify blocked reactions. Applying PSAMM to 57 models collected from current literature, we demonstrated how the software can be used for managing and simulating metabolic models. We identified a number of common inconsistencies in existing models and constructed an updated model repository to document the resolution of these inconsistencies.

## Author Summary

The broad application of genome-scale metabolic modeling has made it a useful technique for tackling fundamental questions in biological research and engineering. Today over 100 models have been constructed for organisms that carry out a diverse array of metabolic activities spanning all three kingdoms of life. These models, however, have been curated independently following different conventions. The maintenance of model consistency has been challenging due to the lack of consensus in model representation and the absence of integrated modeling software for associating mathematical simulations with the annotation and biological interpretation of metabolic models. To solve this problem, we developed a new software package, PSAMM, and a new model format that incorporates heterogeneous, model-specific annotation information into modular representations of model definitions and simulation settings. PSAMM provides significant advances in standardizing the workflow of model annotation and consistency checking. Compared to existing tools, PSAMM supports more flexible configurations and is more efficient in running constraint-based simulations. All functions of PSAMM are freely available for academic users and can be downloaded from a public Git repository (https://zhanglab.github.io/psamm/) under the GNU General Public License.

## Introduction

The GEnome-scale Models (GEMs) of metabolic networks have broad applications in biological research and engineering [1]. Models have been developed for organisms of all three kingdoms of life [2–5] and have been used to simulate a wide variety of metabolic processes, such as photo- and chemo-autotrophic carbon fixation [6,7], fermentation [8], and the production of specific organic compounds [9]. GEMs can be applied in theoretical research to predict gene essentiality [10,11], simulate the thermo-tolerance of bacterial strains [12], and study the structural and functional evolution of metabolic networks [13]. They can also be used in practical studies to identify drug targets [14,15], illustrate the mechanism of human diseases [16], and to optimize the production of compounds of industrial significance [17–19]. By connecting genome annotations with the mathematical simulation of reaction networks, GEMs are particularly applicable for integrating heterogeneous datasets from high-throughput studies [20], such as the profiles of transcriptional regulation [21,22] and measurement of carbon isotope labeling [23].

Specialized MATLAB toolboxes have been released over the past two decades to support the mathematical simulations of metabolic networks. The COBRA Toolbox is a collection of widely used, open source tools. It includes diverse implementations of constraint-based modeling algorithms and has attracted a large number of user contributions from the modeling community [24]. This toolbox, however, is restricted to the MATLAB environment and requires users to maintain paid licenses from MathWorks Inc. The COBRApy software is a more recent implementation of the COBRA Toolbox functions using the Python programming language and Jython, a Java implementation of Python [25]. Like the COBRA Toolbox, COBRApy is released as a toolbox rather than a software package. Therefore, knowledge about the Python programming language is required for users to efficiently set up operations in COBRApy.

Other tools have been developed to support the annotation and visualization of metabolic networks. ModelSEED is a web-based platform that supports automated reconstruction of metabolic models from genome annotations [26,27]. It links protein functions with an internal reaction database and is associated with the SEED functional annotation database and the RAST genome annotation pipeline [28]. In contrast to the COBRA Toolbox, this platform is focused on the reconstruction instead of the mathematical simulation of GEMs. The automated pipeline of ModelSEED permits direct construction of a draft model from genome annotation. However, the draft model still requires extensive manual curations, and manually editing the draft models is not currently supported [27]. The RAVEN Toolbox supports semi-automatic reconstruction and visualization of genome-scale models [29]. It uses information from the KEGG database [30] and, similar to the COBRA Toolbox, can only be applied under the MATLAB environment. Finally, Pathway Tools is another software package that supports pathway annotation and visualization [31]. It uses pathway information from the MetaCyc database [32] and has recently been extended to include functions for flux balance analysis. Unlike the open source software mentioned above, Pathway Tools is released under a restricted license agreement, but it is free to use for academic users.

Despite the technological advances in supporting individual steps of network reconstruction and mathematical simulation, challenges remain in maintaining the quality of metabolic reconstructions and in associating the representation of mathematical problems with the annotation and biological interpretation of metabolic models. Human intervention and iterative manual examinations are required in various steps, such as defining biomass functions, assigning reaction directionality, establishing boundary conditions, and filling novel reaction gaps [26,33]. Frequently, these processes are carried out either manually or using customized tools that were developed by individual model curators. However, due to the absence of standardized procedures for quality checking and version tracking of iterative model annotations, the curation and manual editing of metabolic models are prone to introducing inconsistencies [34]. These inconsistencies may lead to false predictions of model viability, and as demonstrated in a recent study, may hinder the evaluation of new modeling approaches [35–37].

Another problem that prevents the effective curation and consistency checking of GEMs is the disassociations between mathematical and biological representations of model metadata. Despite a broad adoption of the SBML format, it is not designed to incorporate detailed annotations of the genes, reactions, compounds, and pathways. Two strategies have been implemented to help address this limitation. The first is to use user-defined, model-specific or toolbox-specific tags in SBML files (e.g. the <notes> tag in COBRA-compliant SBML format). These tags are not part of the standard SBML specifications and are not recognized by the standardized SBML parsers (e.g. libSBML [38]). The second strategy employs tables of annotation data. These tables are customized to represent specific models, but they are disconnected from the simulation problems defined in SBML files, and an automated parsing of these tables is impossible due to the lack of convention in table organizations. In either case, the mathematical representation of simulation problems is separated from the biological annotation of model components. As a result, the consistency between genome annotation and the definition of simulation settings have to be maintained by individual model curators. This again is prone to introducing misrepresentations in GEMs.

An additional disadvantage of the SBML format is the lack of modularity in network representations. The SBML definition of GEMs is composed of two major lists: *listOfSpecies*, which defines all of the metabolites in a GEM; and *listOfReactions*, which defines all of the reactions and their simulation settings in a single modeling state, including flux bounds, flux values, and objective coefficients. Since the definition of simulation settings interleaves with the definition of static reaction features, SBML does not support reusing the static model definitions. Anytime a new simulation is released on the same base model, information about the static features of metabolites and reactions will need to be duplicated in a new SBML file. Hence, the SBML format is not compatible with a modular organization of modeling components because it mixes the static model properties with the dynamic simulation settings.

Here, we present a Portable System for the Analysis of Metabolic Models (PSAMM), an open source software package that was implemented to support the iterative curation of GEMs by connecting model annotations with mathematical simulations. A novel model format was developed in PSAMM using the YAML language to integrate detailed annotations of model components and to provide flexibilities in both the data format (i.e. support direct referencing of annotation tables or the YAML-based list format) and the data content (i.e. support parsing of both standardized and model-specific data fields). The new YAML format assembles model definition and simulation settings into independent and reusable modules (S1 Text). By reducing the amount of characters used for data structure definition, it streamlines the content of the real data and enables tracking and management of GEMs with conventional line-based version control systems like Git [39]. Applying PSAMM, we constructed an updated repository of 57 published models using the modular representation of the YAML format, and we further demonstrated the importance of model formatting and consistency checking through identifying and correcting potential inconsistencies in existing GEMs.

## Results

### Comparison of PSAMM to Existing Tools

PSAMM was developed as an integrated software package that supports calling simulation functions using simple one-line commands. This distinguishes PSAMM from existing metabolic modeling toolboxes, which require the users to go through individual steps of model loading, linear programming solver selection, model optimization, and simulation results mapping. An example of PSAMM commands is given (S1 Fig), in which a thermodynamically constrained flux balance analysis (tFBA) was performed through a simple one-line command. The result of the analysis contains metadata about the model name, model version, objective function, reaction fluxes, and reaction equations. In contrast, it takes three steps to set up a tFBA problem in COBRA, and the output is presented in a complex data structure that is dissociated from the biological interpretations of the model. The PSAMM implementations were also much more efficient than the COBRA toolbox functions. While it took only 7.15 seconds to solve a tFBA problem in PSAMM, the same process on the same model took 194.73 seconds in COBRA.

**Fig 1:**
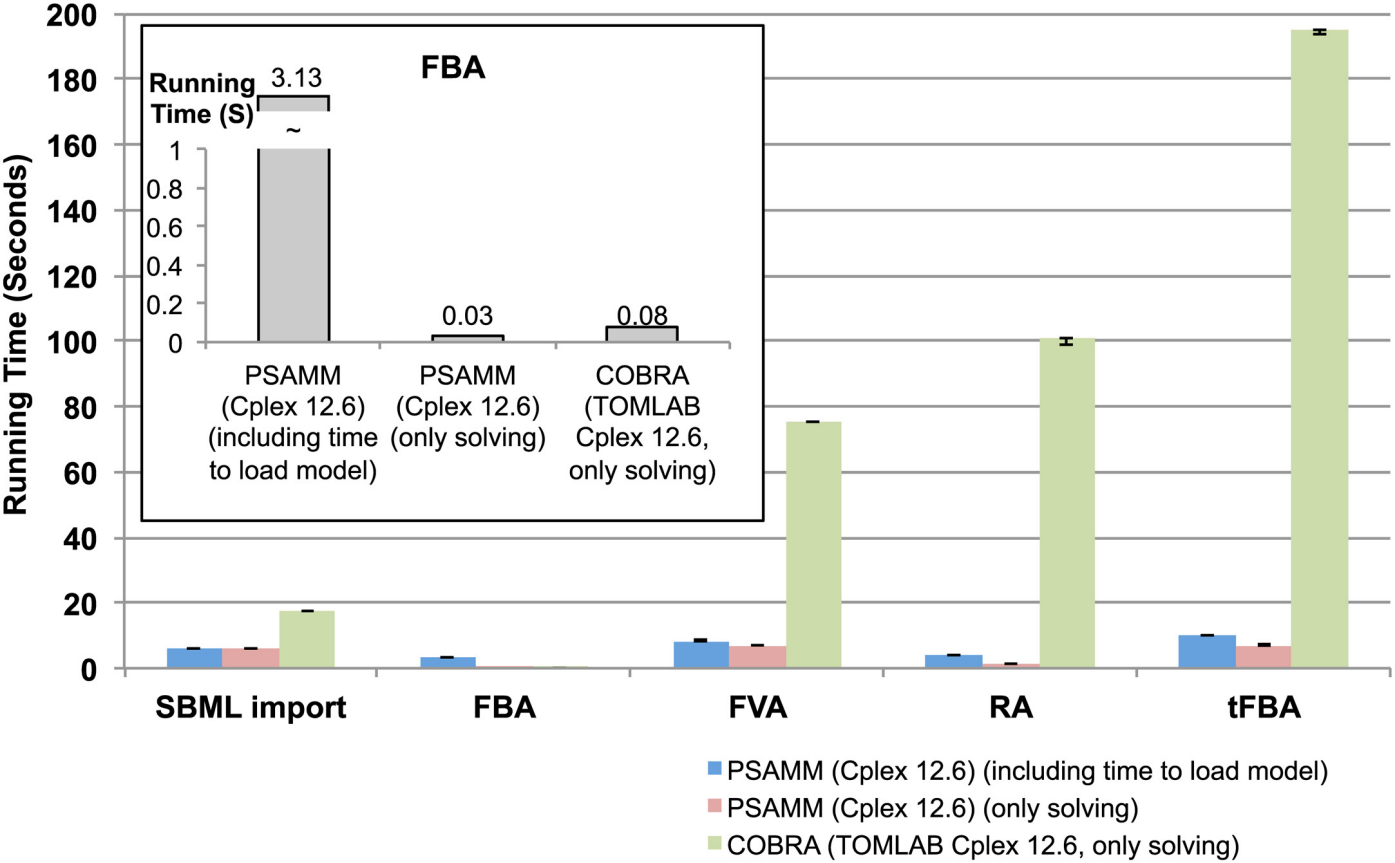
Comparing the running time of different functions in PSAMM and COBRA. The running time for both the PSAMM overall function (blue) and the PSAMM problem-solving steps (red) were calculated, and the running time for COBRA only included the problem-solving step (green). Each value represents a median of seven simulation runs using the same specifications, and the error bars indicate the 25th and the 75th percentiles.

A comparison of the functions in the PSAMM software package with the functions in the COBRA and RAVEN toolboxes is provided in the S1 Table. Besides the availability of diverse functions and implementations of model import/export, model checking, and constraint-based analysis, a unique feature of PSAMM is the availability of easily accessible help information that assists the users in selecting program functions and parameter values. This feature is enabled because PSAMM integrates the various functions under a common framework of two universal programs, *psamm-model* and *psamm-import*. The help information can be accessed in PSAMM by adding the *-h* or --*help* option following any program functions listed in the S1 Table. In contrast, RAVEN and COBRA do not provide an integrated interface to the available functions but instead require the users to know the names of individual functions in order to retrieve help information. Moreover, to systematically evaluate the efficiency of PSAMM implementations, we recorded the running time of common operations in PSAMM and COBRA on the *Escherichia coli* model iJO1366 [40] (Materials and Methods). The results suggested that PSAMM is overall much more efficient than COBRA (Fig 1). For the computationally intensive functions like flux variable analysis (FVA), robustness analysis (Robustness), and thermodynamically constrained flux balance analysis (tFBA), PSAMM ranges from 9 to 25 times faster than implementations in the COBRA toolbox. For the FBA function, PSAMM appears to have an overhead of about 3 seconds on top of the time required for the problem-solving step (Fig 1 inset). This overhead is for the reading of YAML model files, which is also included in calculating the running times of FVA, Robustness, and tFBA, but solving FBA problem in PSAMM (0.03 seconds) is slightly more efficient than the same step in COBRA (0.08 seconds). The running time for RAVEN was not plotted in Fig 1 because many of the functions are unavailable in the RAVEN toolbox (S1 Table). A detailed record of the running times for available functions in COBRA, RAVEN, and PSAMM is provided in the S2 Table. Overall, PSAMM is more efficient than the COBRA and RAVEN toolboxes in carrying out constraint-based analysis of GEMs.

### PSAMM Implementation

**Fig 2:**
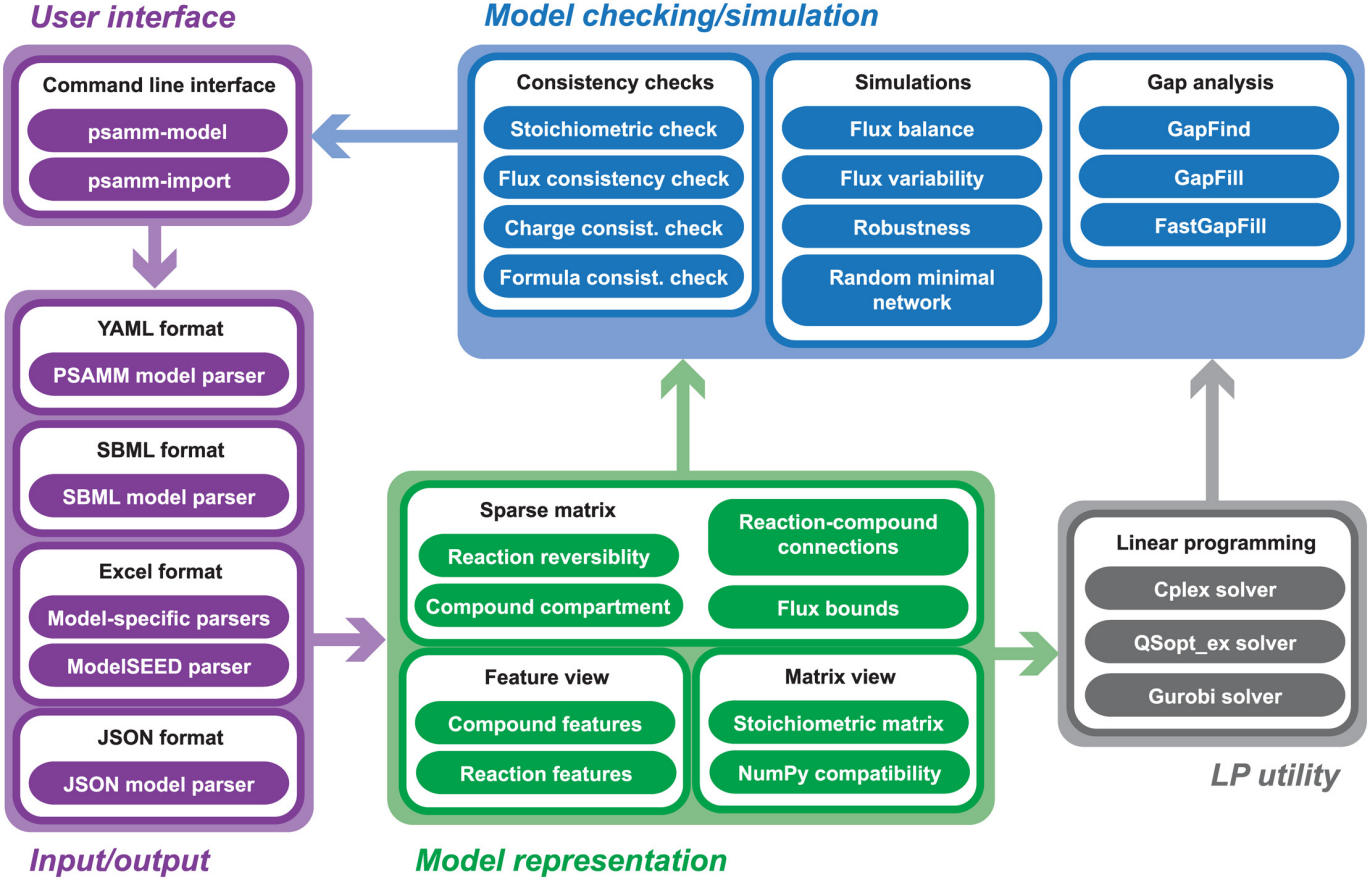
Overview of the internal workflow in PSAMM. The five main components include: (1) user interface, (2) model input/output, (3) model representation, (4) linear programming utilities, and (5) model checking/simulation. Connections among these components form the internal workflow of PSAMM.

The PSAMM software package includes five main components: user interface, model input/output, internal model representations, model checking/simulation, and linear programming utilities (Fig 2). These components are interconnected with one another to form the internal workflow of model importing and model optimizations in PSAMM. Below, we provided a detailed description of these functions.

#### User interface

The user interface was implemented as two command-line programs that provide entry points to PSAMM functions. The first program, named *psamm-model*, is the main interface for model consistency checking, constraint-based simulation and automatic gap analysis. The commands that can be specified with *psamm-model* encapsulate both commonly used metabolic modeling procedures (e.g. *fba*, *fva*, and *gapfill*) and PSAMM-specific procedures, such as *masscheck*, *fluxcheck*, *randomsparse*, *search*, and *console* (Table 1 and S1 Table). The second program, named *psamm-import*, facilitates the recognition and conversion of multiple GEM formats, including the SBML format that is most widely used as a convention for model distributions in current literature, the Microsoft Excel format that is frequently used to store the annotation of compound and reaction features, and the JSON format that is developed within the openCOBRA project. The *psamm-import* program converts the SBML, Excel, and JSON files into a YAML-based format that was designed as a new convention in PSAMM for integrating user-defined annotations with mathematical simulations of metabolic models (S1 Text). The *psamm-model* and *psamm-import* programs interface directly with the model input/output module, which permits the import of model files into internal model representations and the export of model information into standard SBML, YAML, and Excel files.

**Table 1:** List of commands supported in the *psamm-model* program. Additional parameters can be specified for the commands, for example to select a specific implementation among multiple algorithms, enable/disable thermodynamic constraints, or select linear programming solvers, *etc*. Details about these parameters are available through the *-h* or --*help* options for each command.

	Command	Function
**Model Consistency Checking**	*masscheck*	Stoichiometric (mass) consistency check
	*fluxcheck*	Check for blocked reactions
	*formulacheck*	Chemical formula balance check
	*chargecheck*	Charge balance check
**Constraint-based Simulations**	*fba*	Flux balance analysis
	*fva*	Flux variability analysis
	*robustness*	Robustness analysis
	*randomsparse*	Find random minimal network
**Gap Analysis**	*gapfill*	Gap analysis using the GapFind and GapFill algorithms
	*fastgapfill*	Fast gap analysis using the fastGapFill algorithm
**Model Interfacing**	*search*	Looking up the detailed features of specific compounds or reactions.
	*console*	Opening an interactive Python or iPython session with the model loaded in memory. Providing a programming interface for the customized inspection and analysis of GEMs.
	*sbmlexport*	Export the model to SBML format.
	*excelexport*	Export the model to Excel format.

#### Model input/output

The model input/output module includes functions for importing model files in the SBML, Excel, JSON and YAML formats, and exporting models in the YAML, Excel, and SBML formats. PSAMM supports importing both the standard SBML specifications and the COBRA-compliant SBML format [24], and the parsing of model files was based on a built-in SBML parser instead of relying on external dependencies like the *libSBML* [38]. This reduction of external dependencies can simplify the installation process and hence increase the portability of PSAMM. Moreover, the development of a built-in parser enables PSAMM to recognize model-specific data entries that are not part of the standard SBML specification. Using the built-in parser, users can check if a model is strictly compliant with the SBML specifications and import it by setting the *sbml-strict* option in the *psamm-import* program. The *sbml-strict* option will only check for parameters defined in the standardized SBML specifications [41]. However, to load constraint-based modeling parameters, such as the reaction bound parameters defined in the COBRA-compliant format, the users can use the more flexible *sbml* option. This later option is also extended to automatically resolve some common inconsistencies in existing models, such as permitting decimal numbers in a SBML level that supports only integers in stoichiometry or recognizing a special suffix “*_b*” that indicates exchange compounds (Table 2). When a potential inconsistency is resolved automatically, *psamm-import* will print a warning message so that users can examine if the SBML parsing was performed correctly.

**Table 2:** List of models that had inconsistencies in SBML syntax.

Model	Organism	Inconsistencies
AbyMBEL891	*Acinetobacter baumannii*	Decimals in stoichiometric values is not supported in Level 1 SBML specification
SpoMBEL1693	*Schizosaccharomyces pombe*	Decimals in stoichiometric values is not supported in Level 1 SBML specification
iJN746	*Pseudomonas putida*	Undefined compound references in the biomass reaction
iNJ661m	*Mycobacterium tuberculosis*	COBRA-compliant, using a special suffix “*_b*” instead of defining “*boundaryCondition*” based on SBML standard
iNJ661v	*Mycobacterium tuberculosis*	COBRA-compliant, using a special suffix “*_b*” instead of defining “*boundaryCondition*” based on SBML standard
iSR432	*Clostridium thermocellum*	COBRA-compliant, using a special suffix “*_b*” instead of defining “*boundaryCondition*” based on SBML standard
iTZ479	*Thermotoga maritima*	COBRA-compliant, using a special suffix “*_b*” instead of defining “*boundaryCondition*” based on SBML standard
iTH366	*Plasmodium falciparum*	COBRA-compliant, using a special suffix “*_b*” instead of defining “*boundaryCondition*” based on SBML standard
iRC1080	*Chlamydomonas reinhardtii*	Inconsistencies between reversibility and “LOWER_BOUND” settings
iRS1563	*Zea mays*	Inconsistencies between reversibility and “LOWER_BOUND” settings
iRS1597	*Arabidopsis thaliana*	Inconsistencies between reversibility and “LOWER_BOUND” settings
iRsp1095	*Rhodobacter sphaeroides*	Nutrient not provided in the simulation environment
iMA871	*Aspergillus Niger*	Accumulation of an artificial *M_BIOMASS* compound
iMA945	*Salmonella enterica*	Mislabeling of compound compartments
iSyn731	*Synechocystis sp*. PCC 6803	Potential inconsistencies in model conversions

Besides SBML files, many GEMs were published in the format of Microsoft Excel tables that contain the definition of compound and reaction features. However, so far no standard has been specified for the Excel format. To incorporate these models, PSAMM provides a customized interface that enables importing of model-specific Excel tables and the Excel tables generated from automated model reconstruction pipeline in ModelSEED [26]. Additionally, PSAMM supports the import of JSON models either from local files or from the BiGG database [42]. The import functions for both JSON and Excel formats were implemented in the *psamm-import* program, which converts models into the PSAMM-specific YAML format (S1 Text).

#### Internal model representations

The PSAMM internal model representations provide three distinct data structures for the organization of model information. At the hub is a sparse matrix, which stores a global mapping between compounds and reactions. The sparse matrix can include information regarding stoichiometry, compound compartments, reaction reversibility, and flux limits. It provides a central framework for the management and analysis of metabolic models and can be queried into two data structures that support the implementation of PSAMM functions. In the first data structure, a feature view can be created to incorporate additional information about the model metadata, including compound features (e.g. formulas, charges, etc.) and reaction features (e.g. names, subsystem classifications, equations, etc.). In the second data structure, a matrix view can be initiated to represent a full-scale stoichiometric matrix. The diverse data structures in PSAMM enables the integration of heterogeneous data regarding the user-defined model annotations as well as the mathematical simulations of GEMs. These internal model representations allow for the formulation of modeling problems by interfacing with the linear programming (LP) utility and the model checking/simulation modules.

#### Linear programming (LP) utilities

The LP utilities were implemented as an internal component that supports the definition of LP problems and the parsing of LP solutions. Three LP solvers are supported in the PSAMM utilities: the IBM ILOG Cplex Optimizer, the Gurobi Optimizer, and the QSopt_ex Rational Solver (Materials and Methods). While Cplex and Gurobi provide approximated solutions for both LP and mixed integer LP (MILP) problems, QSopt_ex computes exact solutions only for LP problems. The QSopt_ex Rational Solver is supported because it has recently been suggested to provide a more accurate solution for constraint-based modeling problems [35]. Applying both the approximated solvers and the exact solvers under the same framework of PSAMM model representations will enable a standardized comparison of these two approaches. The LP utilities form a backend module in PSAMM that communicates with the Application Programming Interface (API) of external solvers and provides LP solutions to the model checking/simulation functions.

#### Model checking/simulation

The model checking/simulation module includes diverse user functions for model consistency checking, constraint-based simulation, and gap analysis (Fig 2). It was developed based on the internal feature representation of GEMs to support the listing of simulation results along with the biological annotations. Thus, mathematical simulation results can be analyzed under a user-friendly interface that permits the direct association of the numeric values with the biologically meaningful representations of biochemical equations (S1 Fig).

The constraint-based simulation functions include flux balance analysis (FBA) [43,44], flux variability analysis (FVA) [45], robustness analysis [46], and random minimal network analysis [47] (Table 1 and S1 Table). Each function was implemented to support two universal options: --*loop-removal*, which determines whether thermodynamic constraints should be applied to the LP problem, and --*solver*, which allows the users to select an LP solver while running the simulation problem. The random minimal network analysis (*randomsparse*) is a unique function in PSAMM that is not available in any existing toolboxes. Each round of the *randomsparse* performs a series of random gene deletions, which result in a minimal metabolic network where all genes are essential for biomass production. The median running time for 100 rounds of *randomsparse* analysis on iJO1366 [40] is 1,664 seconds (S2 Table). In case gene association information is not available, the command can also be applied directly to reactions, which results in a minimal network where all reactions are essential. The median running time for 100 rounds of the reaction-based *randomsparse* analysis on iJO1366 is 3,524 seconds (S2 Table). Hence, each round of the successive random deletions takes less than or close to 30 seconds.

The consistency checking and gap analysis functions were set up to assist with the model curation and to examine stoichiometric, flux, charge, and formula consistencies of individual reactions (S1 Table). While the stoichiometric check can provide a global estimation of whether all reactions in a model are mass balanced (Materials and Methods), the charge and formula checks can examine the compound element and charge balances in individual reactions and can only be applied when the compound charge and formula is defined in the GEM. The flux check function was implemented to identify reactions that are unable to carry a non-zero flux under any modeling constraints. It can be used to reveal disconnected areas of the model and to ensure unbiased interpretation of the model simulations (Materials and Methods). The gap analysis functions were implemented based on two existing algorithms, GapFind/GapFill [48] and fastGapFill [49]. Both functions can recommend a list of new reactions that should be added into a given model. Depending on the scope of information included in the YAML reaction database (S1 Text), the prediction of gap reactions may be based on a list of enzymatic reactions in the broader reaction database, or artificial exchange reactions that point out the missing links between gap compounds and the modeling environment.

### The PSAMM YAML Format of Model Representation

**Fig 3:**
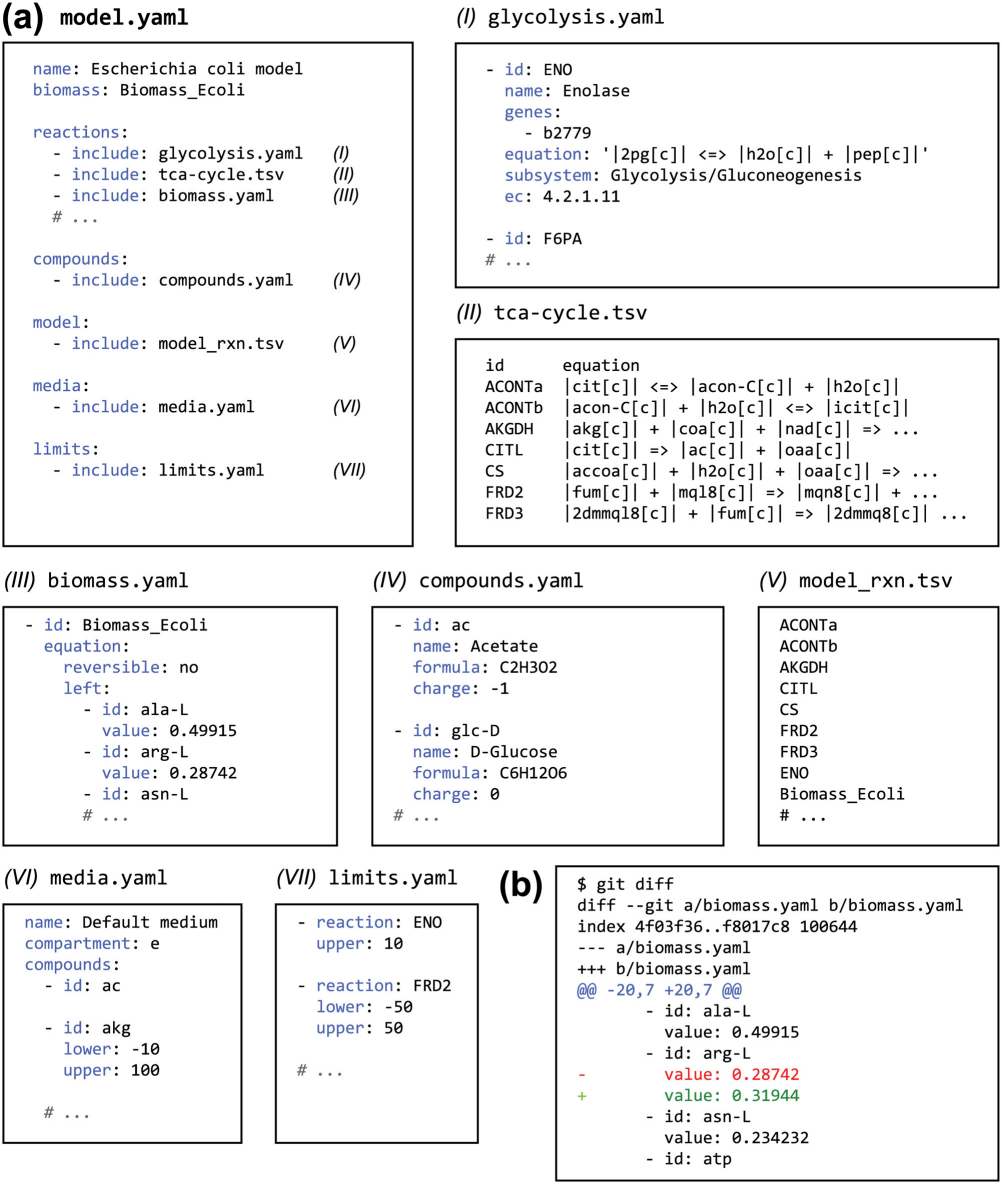
An illustration of the PSAMM YAML format. (a) This diagram shows an example of the YAML model format, which includes a central model definition (model.yaml) and multiple annotation files. Each box indicates a file with a possible filename indicated above the box, and the text within is a snapshot of the file content. (b) An example showing how changes can be tracked in a PSAMM YAML file (biomass.yaml) using the Git version control system in command line. The text highlighted in red indicates the stoichiometry of the compound arg-L in an old version of the biomass function, while the text highlighted in green indicates the updated value in a new version of the model. Additional examples of applying Git version control on the YAML format are provided in the supplemental materials (S2–S5 Texts).

A new model format was implemented in PSAMM based on YAML, a serialization language that is designed to support both computational parsing and human readability of complex data structures [50]. The YAML format has several new features that distinguish it from the SBML format of constraint-based metabolic models. First, while SBML strictly couples the model definition with a single simulation condition, YAML allows the users to freely combine model definitions with simulation setups in a modular form. Hence, a model component can be reused in multiple simulation setups simply by using the *include* function (Fig 3 and S1 Text). In contrast, to publish the simulation results of multiple modeling conditions in the SBML format, one has to build duplicated instances of the entire model definition to introduce variability in simulation setups. Second, while SBML is suboptimal in working with the widely-used version control systems like Git [51], the YAML format is fully compatible and supports tracking of model edits with these systems. This is because YAML, by applying line breaks and whitespaces as meaningful structural information, can be tracked in a way similar to programming scripts using the longest common sequence (LCS) algorithm that is commonly used by Git [52]. In contrast, SBML does not have a convention of breaking lines, and therefore the LCS line-based version tracking will have difficulties in pinpointing the exact location of changes in the SBML files. Despite the development of new algorithms to support the comparison of SBML model files, the efficient and reliable tracking of changes in the SBML format is still challenging [34,51]. Third, while SBML relies on the verbose representation of markup tags, the YAML format minimizes the amount of characters used for marking up the structure of the data to streamline the integration of complex data (Fig 3 and S1 Text). Finally, while the parsing of SBML files requires knowledge of predefined markup tags to capture specific data fields, the parsing of YAML files can be achieved by the recognition of line-based data structure rather than relying on the identification of specific markup tags. For example, some features like the compound formula and reaction EC numbers are not an integral part of standardized SBML specifications [41], so they will be invisible to standard SBML parsers (e.g. libSBML [38] or JSBML [53]) even when they are included in a SBML file with user-defined markup tags. However, the same information can be easily represented in YAML files. A standard YAML parser will have no problem recognizing user-defined, non-standard data domains and subsequently integrate it into the presentation of metabolic models. Overall, the YAML format is an optimized solution for connecting manual curation with the mathematical simulation of GEMs.

The PSAMM YAML-based representation of GEMs includes the model name, biomass function, compound/reaction database(s), model reaction lists, reaction flux limits, and growth media. A flexible infrastructure was designed that permits the integration of model definitions either within a centralized file (e.g. model.yaml) or in additional files using the *include* function (Fig 3a). In an example shown in Fig 3a, the reaction database was divided into multiple files based on pathway classification, and both the YAML format (e.g. glycolysis.yaml and biomass.yaml) and the tab-delimited table format (e.g. tca-cycle.tsv) were illustrated. Similarly, the compound annotations can also be represented as YAML files (e.g. compound.yaml) or tab-delimited tables. The reaction and compound files can be used to define a broader database of all possible reactions with additional entries beyond the scope of a given model. For example, when running the *gapfill* or *fastgapfill* functions, users may include a broader reaction database that contains enzymatic reactions that can be potentially applied to close network gaps. In such cases, the model file (e.g. model_rxn.tsv) is used to identify a subset of reactions that are included in the model definition. The other reactions in the broader database will be ignored in model simulations and will only be probed in *gapfill* or *fastgapfill* to predict potential gap filling reactions. Finally, to define the constraints for metabolic simulations, the media file (e.g. media.yaml) is used to identify the exchange reactions, and the limits file (e.g. limits.yaml) is used to set the flux limits for internal reactions. The limits file is defined only when the flux bounds of an internal reaction deviate from the defaults in PSAMM, which automatically assigns a conventional boundary of [−*x*,*x*] for reversible reactions and [0,x] for irreversible reactions, where the value x is assigned using an option named *default_flux_limit* in the model.yaml file. When *default_flux_limit* is not assigned, PSAMM will use a default value of x = 1000 to define reaction bounds (S1 Text). This feature permits further streamlining of the YAML format to reveal model specific simulation setups. Although PSAMM supports references to model files with user-defined file names that are saved in distributed locations in the file system, it is recommended that users save all files of an individual model within a dedicated directory to facilitate model organization and maintenance. The users may also choose to combine all the model information into a single YAML file by replacing the *include* functions with the actual content of the data files. However, the *include* functions are recommended to maintain modularity of model definition and simulation settings in the YAML representation.

Using the YAML format, annotations of compound and reaction features can be further divided into multiple files that represent logical divisions of cellular compartments or metabolic pathways. Moreover, The annotations can be represented in either a YAML format or a tab-delimited table format. These flexibilities can enable the manual curation of model files and make the model components portable among different simulation conditions. The line-based text representation of YAML files also makes it compatible with broadly-used version control systems like Git, and the YAML format is especially efficient at tracking changes in large reaction equations, such as the biomass function and the protein, RNA, and lipid synthesis functions (Fig 3b). In contrast, the Excel format is not compatible with version control due to the binary form, and the lack of a standard model definition in Excel files also detracts from its suitability as a GEM format. The SBML files, in spite of a well-defined data structure, do not use line breaks or white spaces as a part of the data structure and hence are suboptimal in working with Git [34]. For example, each list item in the SBML file is frequently represented as a single line that contains multiple data entries describing this item. This will render the version control in Git ineffective because changes on any one entry will cause the version tracking system to highlight an entire line of multiple data entries, and it is difficult for a user to pinpoint the exact location of the change within a long line of data. Moreover, since the markup tags are included as a part of the data presentation, changes in the markup tag definitions during the periodical updates of the SBML specifications will show up in the tracking even though there is no change in the model data.

### Examining Model Consistencies Using PSAMM

To test the application of PSAMM in supporting model annotations, we applied the software to 57 published GEMs collected from current literature or open-source model releases (S3 Table). These included the RECON2.04, which was released in the format of a MATLAB data file. All models were first converted to the PSAMM-specific YAML format and then analyzed individually using the stoichiometric and flux checking functions. FBA simulations were also performed using designated biomass function to verify if the published descriptions of model viability can be replicated. When inconsistencies occurred, the causes were identified by manually inspecting the corresponding models. Overall, PSAMM revealed several types of inconsistencies among existing models. These included inconsistencies in the syntax of SBML files, the annotation contents of Excel tables, the stoichiometric balance of metabolic reactions, and the connectivity of metabolic reactions. Below, we provide an overview of the existing inconsistencies identified by PSAMM.

#### Inconsistencies in SBML syntax

The Systems Biology Markup Language (SBML) community has published three different specifications (levels) of the SBML format. Of the 57 GEMs we analyzed, 56 were released as SBML files, including two level-1 models (AbyMBEL891 [54] and SpoMBEL1693 [3]), three level-3 models (iMM1415 [55], iMO1056 [56], and RECON1 [57]), and 51 level-2 models as defined in their SBML file headings. The SBML models were converted to YAML format using the *psamm-import* program. First, the *sbml-strict* option was used to check if the model follows strict SBML specifications. Then, the *sbml* option was used to incorporate COBRA-compliant variations in SBML syntax. Finally, an FBA simulation was performed using biomass production as the objective function to examine the viability of each model.

Using the *sbml-strict* option of *psamm-import*, inconsistencies were found in the SBML syntax of 15 GEMs (Table 2). These included inconsistencies both in the model specification and in the definition of simulation setups. Three models were not strictly compliant with the standardized SBML specifications, including two models that contain decimal numbers in a SBML level that supports only integers in stoichiometry (AbyMBEL891, SpoMBEL1693) and one model that contains undefined compound species (i.e. *cardiolipin[c]*) in the biomass function (iJN746 [58]). With the *sbml* option, the former inconsistencies were resolved by permitting decimals in stoichiometry for all SBML levels, and the latter was resolved by automatically removing undefined compound species in reaction equations. In both cases, warning messages were given by *psamm-import* so the users can be aware of these modifications. To ensure a more accurate parsing of iJN746, we also manually examined the model to look for potential production pathways of the undefined cytosolic *cardiolipin[c]* compounds. This manual curation revealed that the missing compounds were actually mislabeled in their compartment settings: while the synthesis functions occurred in periplasmic space, the biomass function referred to a non-existing cytosolic copy. Since experimental results suggest that the synthesis of cardiolipin compounds occurs in periplasm [59,60], we decided to manually modify the biomass function of iJN746 by changing the compartment definition of the *cardiolipin[c]* compounds into periplasm (i.e. *cardiolipin[p]*) (S2 Text). After the manual correction, iJN746 was able to produce a non-zero biomass flux.

Another five models, iNJ661m [61], iNJ661v [61], iSR432 [62], iTZ479 [13], and iTH366 [63], initially failed to generate a non-zero biomass flux under FBA when loaded with the *sbml-strict* option. A close examination of these non-viable models revealed additional inconsistencies in SBML syntax, where an attribute named “*boundaryCondition*” was missing. This was fixed by using the *sbml* option of *psamm-import*, which is compatible with the COBRA convention and can automatically create exchange reactions for compounds with a special suffix (i.e. the “*_b*” suffix that indicates boundary compounds).

Additionally, five models had inconsistencies that needed to be manually examined before a viable simulation could be reproduced. The models iRC1080 [64], iRS1563 and iRS1597 [65] contained reactions that were marked irreversible (meaning that a reaction should only progress in the forward direction) but at the same time had the “*LOWER_BOUND*” parameter set to a negative value (meaning that the reaction should be able to progress in the reverse direction). This reflected inconsistencies between the COBRA-compliant format and the SBML standard specifications. To ensure that users can be aware of such inconsistencies, we set up the *sbml* option of *psamm-import* to produce a warning message and by default allowed reactions that had inconsistent reversibility settings to carry fluxes according to the flux bounds specified using the COBRA-compliant format. The model iRsp1095 [66] contained no exchange reaction that would supply nutrients to the simulation environment. This was due to the model defining all exchange reactions in the SBML file as sinks but not sources of boundary compounds. To resolve such inconsistency, we manually set the flux limits of all exchange reactions according to the minimal medium reported in the original publication [66] to permit uptakes of nutrient compounds that lead to the production of non-zero biomass flux (S3 Text). The model iMA871 [67] was not viable because a symbolic compound of the biomass, *M_BIOMASS*, was accumulated as a result of the biomass reaction, which deviated from a steady-state assumption of the FBA simulation. This was resolved by connecting the *M_BIOMASS* compound with a biomass sink flux, which simulates the biomass production of the model (S4 Text).

Finally, two SBML models, iSyn731 [65] and iMA945 [68], were not viable and could not be fixed by simply introducing flexibilities in SBML parsing. The iMA945 model contains a large number of reaction equations that carry duplicated compounds on the opposite sides, which were likely introduced to the model due to the mislabeling of compound compartments. This inconsistency was corrected by importing the model from an Excel file released in the original publication of iMA945. In the Excel file, compound compartments were assigned correctly and there were no duplicated compounds in any reactions. The iSyn731 model was corrected using an updated version of the SBML release downloaded from the original authors’ website (S5 Text). A comparison of the updated version with the original version of iSyn731 revealed a number of changes in reaction directionality and stoichiometric balance, which might be the result of unintentional inconsistencies in the original conversion of the author’s internal model annotations into the SBML format. Hence, this further illustrated the importance of standardized model version tracking and integrated model representations. The YAML files resulting from importing all the above-mentioned SBML models and all the manual corrections were documented in an open-source Git repository at https://github.com/zhanglab/psamm-model-collection.

#### Inconsistencies in excel files

As an important part of a standard model release, most of the published GEMs were supplemented with Excel files. Compared to the SBML format, the Excel tables usually include more extensive metadata, such as the compound and reaction features, into the model definition. However, so far there is no standardized definition of the Excel format. Hence, the Excel import function was implemented individually for specific models. In the PSAMM input/output module, customized Excel parsers were created based on a supervised process, in which the content of Excel tables were examined and model specific scripts were developed to parse out the complete information that defines the nine Excel models we analyzed (S3 Table). FBA simulations were also performed on the biomass functions to examine the viability of each Excel model. In cases where FBA resulted in a zero biomass flux, we further examined the non-viable models using a combination of manual curations and comparisons to the corresponding SBML releases. This analysis uncovered a number of inconsistencies in the Excel files. For example, in the reaction worksheet for model iMA945 [68], the columns of reaction equations and gene identities were merged for a subset of entries, causing a column shift in these entries. By correcting the alignment of table columns, the model was parsed successfully using the customized Excel parser for iMA945. Another example of inconsistencies involved the misspelling of compound identifiers. The reaction worksheet of model iMR1_799 [69] contained an invalid compound “*aaacoa*” in the reaction equation of acetyl-CoA C-acetyltransferase, which as revealed by comparing to the SBML release of the same model, is a misspelled identifier of the compound Acetoacetyl-CoA (“*aacoa*”). Similar inconsistencies were found in other models and were specifically corrected in the construction of model-specific parsers to map the misspelled compounds to corrected names. These inconsistencies have highlighted the difficulty in preventing unintended edits to annotation tables since the observed column shifts and misspellings are likely a result of the manual insertion of compound or reaction entries in the Excel format. This problem can be resolved with the PSAMM YAML format, which permits the direct incorporation of annotation tables into the model using a simple *include* function (Fig 3 and S1 Text). By submitting YAML models to the Git version control, all edits to the table will be recorded and changes that render the model non-viable can then be pinpointed for correction. Moreover, by maintaining the independence of individual *include* functions, the YAML format effectively incorporates the diverse model metadata that is traditionally included in a scattered set of Excel worksheets into an integrated documentation of GEMs.

#### Stoichiometric inconsistencies

The stoichiometric consistency of all GEMs was analyzed using the stoichiometric consistency checking approaches implemented in the PSAMM *masscheck* function (Materials and Methods). Surprisingly, only 18 out of the 57 models passed the criteria of stoichiometric consistency check, suggesting that most models included reactions that are not mass balanced (S3 Table). This is alarming especially in the context of constraint-based analysis because unbalanced reactions can potentially generate infinite source of materials. For example, the model iKF1028 [70] contains two mutually exclusive reactions, RR00610 and RR08939, which differ only by the presence or absence of two additional *H+* compounds on one side of the reaction equations (Table 3, reactions marked with *). Both reactions cannot occur simultaneously in the same model, otherwise the combination of the two can result in a physiologically impossible overall function that generates or consumes an infinite amount of *H+* compounds at no cost. Running a compound-based stoichiometric check on iKF1028 (S1 Table, command “*psamm-model masscheck*”), we identified two compounds, *H+* and *H+[e]*, as the sources of stoichiometric inconsistency. However, this information is not sufficient for model correction because the compounds *H+* and *H+[e]* occurred in 460 reactions in the model and only a small subset of those were unbalanced. Applying a new algorithm in the PSAMM *masscheck* function (Materials and Methods and S1 Table, command “*psamm-model masscheck --type=reaction*”), a list of 14 unbalanced reactions was identified (Table 3), which greatly reduced the number of reactions that required manual examinations. By comparing the number of hydrogen elements on both sides of the 14 reaction equations, the model was revised to fulfill the requirements of stoichiometric consistency. The *masscheck* function can be applied for the identification of unbalanced reactions in other models following the same procedure as demonstrated in the analysis of iKF1028. Unlike the *formulacheck* function, which relies on the definition of compound formulas, *masscheck* can be applied even when compound formulas are not available. This feature is especially useful in the annotation of existing SBML models, as many of the SBML releases do not contain annotation of the compound formulas. When using *masscheck*, users could make expert decisions to exclude reactions (using the --*exclude* option) that are expected to be unbalanced. These may include the exchange and biomass reactions, and the reactions for which the biochemistry is undetermined (i.e. biosynthesis of polymer molecules). Overall, the PSAMM *masscheck* function is optimized to pinpoint the sources of inconsistency among hundreds and thousands of reactions, and it provides flexibility for identifying stoichiometric inconsistencies in both compounds and reactions even when the mass and formula annotations are missing.

**Table 3:** Stoichiometric inconsistencies in iKF1028 [70]. Reaction: the reaction identifiers in the GEM; Equation: the reaction equations; H left/right: the total number of H atoms at the left/right side of the equations; H residue: the differences between the number of H atoms at the left versus the right side of the equations. Two reactions, RR08939 and IR01815, are shown at the bottom of the table, which correspond to the balanced version of the inconsistent reactions RR00610 and IR04287, respectively. Both pairs (marked with * and **, respectively) were present in iKF1028, rendering the overall model stoichiometrically inconsistent.

	Reaction	Equation	H left	H right	H residue
Unbalanced reactions	IR00136	[c]: 2-Oxoglutarate --> Succinate semialdehyde + CO2	4	5	-1
	IR00159	[c]: H2O + Acetyl phosphate --> Orthophosphate + Acetate	5	4	1
	IR00373	[c]: L-Ornithine --> CO2 + Putrescine	13	14	-1
	IR00765	[c]: NADP+ + H2O + L-Valine --> NH3 + NADPH + 3-Methyl-2-oxobutanoate	38	36	2
	IR01585	[c]: D-Glucose 6-phosphate + UDPglucose --> alpha,alpha'-Trehalose 6-phosphate + UDP	33	32	1
	IR04287**	[c]: Uroporphyrinogen III + (2) S-Adenosyl-L-methionine --> Precorrin 2 + (2) S-Adenosyl-L-homocysteine + H+	82	89	-7
	IR04586	[c]: NADH + NH3 + H+ + Pyruvate --> L-Alanine + H2O + NAD+	34	35	-1
	IR09357	[c]: (S)-3-Hydroxy-3-methylglutaryl-CoA --> Acetyl-CoA + Acetoacetate + (5) H+	39	44	-5
	IR10056	[c]: Precorrin 2 + S-Adenosyl-L-methionine --> Precorrin 3A + S-Adenosyl-L-homocysteine + H+	71	64	7
	IR10481	[c]: Aminoimidazole ribotide + HCO3- + ATP --> 5-Carboxyamino-1-(5-phospho-D-ribosyl)imidazole + ADP + Orthophosphate	25	27	-2
	RR00067	[c]: NAD+ + (2) L-Glutamate <==> L-Glutamine + NADH + 2-Oxoglutarate	42	41	1
	RR00610*	[c]: D-Glucose 1-phosphate + (2) H+ <==> D-Glucose 6-phosphate	13	11	2
	RR00692	[c]: D-Fructose 1,6-bisphosphate <==> Dihydroxyacetone phosphate + (4) H+ + D-Glyceraldehyde 3-phosphate	10	14	-4
	RR10479	[c]: 5-Carboxyamino-1-(5-phospho-D-ribosyl)imidazole <==> 1-(5-Phospho-D-ribosyl)-5-amino-4-imidazolecarboxylate	14	11	3
Balanced reactions	IR01815**	[c]: Uroporphyrinogen III + (6) H+ + (2) S-Adenosyl-L-methionine --> Precorrin 2 + (2) S-Adenosyl-L-homocysteine	88	88	0
	RR08939*	[c]: D-Glucose 1-phosphate <==> D-Glucose 6-phosphate	11	11	0

#### Flux inconsistencies

**Fig 4:**
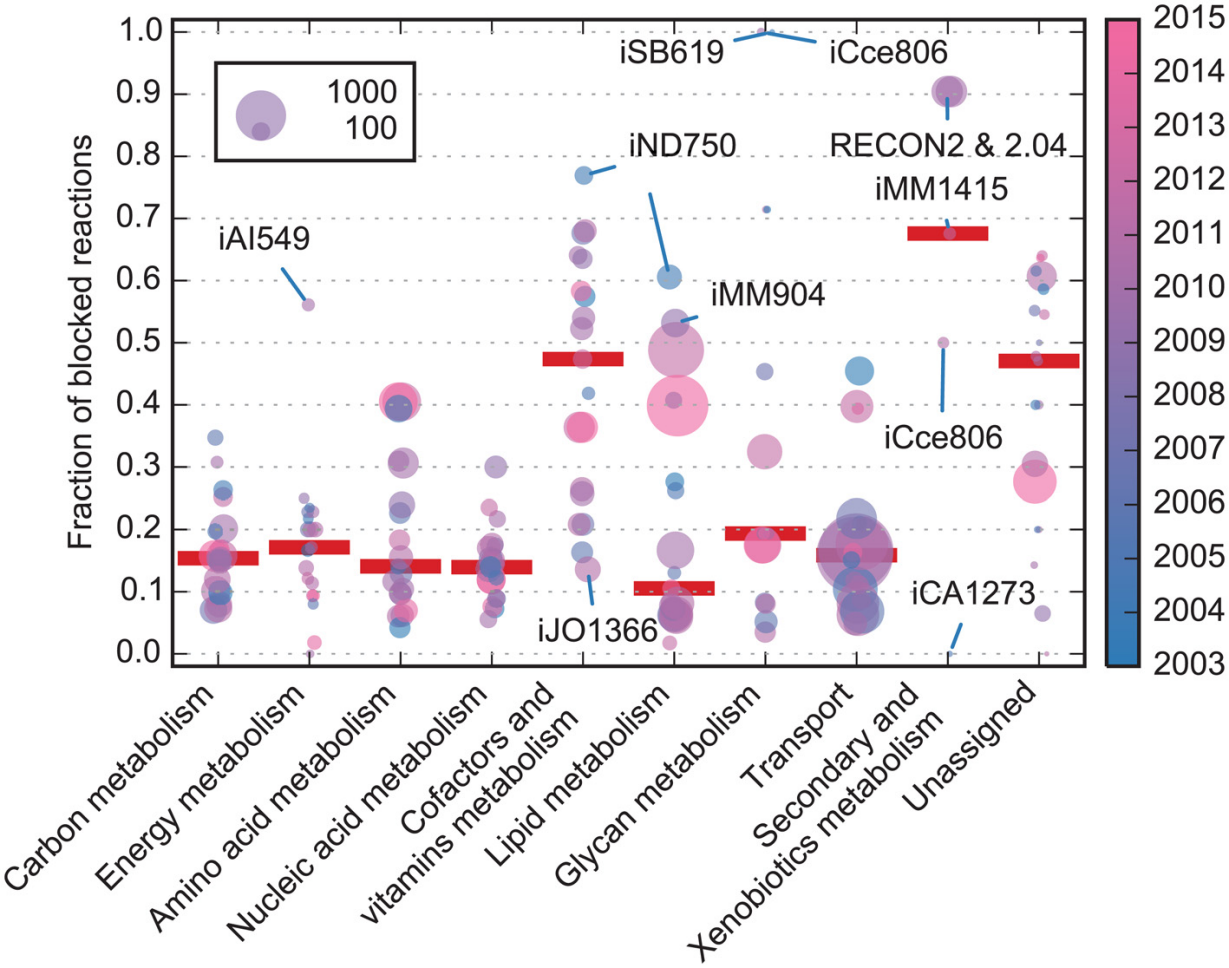
Distribution of blocked reactions in metabolic pathways. The GEMs were represented in each metabolic pathway as a solid circle. The color of the circles corresponds to the year in which a GEM was published (color legend was shown on the right, and the year of publication ranges from 2003 to 2014). The area of the circle is proportional to the total number of reactions in the pathway, and its vertical position indicates the fraction of reactions that are blocked. The median fractions were indicated by a red mark for each pathway, and models discussed in the main text were highlighted.

The PSAMM *fluxcheck* function was applied to all the GEMs to search for flux inconsistent reactions–the reactions that are blocked and unable to carry a non-zero flux under any simulation conditions (Materials and Methods). On average, 30% of all internal reactions in the GEMs were blocked, suggesting that these reactions were topologically disconnected from the rest of the model. We looked into the distribution of blocked reactions among functional subsystems by analyzing a subset of 19 GEMs that contained subsystem annotations in the SBML format (S3 Table). First, subsystem annotations in the GEMs were obtained from SBML files using *psamm-import*, which identified 436 distinct subsystem names from the 19 GEMs. Then, the subsystem names were manually curated and classified into ten metabolic pathways (S4 Table). Finally, the fraction of blocked reactions was calculated within each metabolic pathway, and the median of fractions was identified across all analyzed GEMs (Fig 4).

As a result, most of the metabolic pathways demonstrated high level of flux consistency, and the fraction of blocked reactions had a median lower than 0.2 for seven out of the ten pathways, including four pathways in the central metabolism, two pathways for the metabolism of lipid and glycan, and one pathway of transport reactions. Not surprisingly, the four central metabolic pathways, carbon, energy, amino acid, and nucleic acid metabolism, had the lowest fraction of blocked reactions and were the least variable among different GEMs. This not only reflected their essential roles in biomass production, but also indicated the extent of research that contributed to the complete reconstruction of these pathways. One outlier, however, was the energy metabolism pathway in iAI549 [71], which had 23 blocked reactions out of the 41 total reactions. This was largely due to the lack of certain nutrient sources that drive the energy functions. For example, the model contained two nitrite reductase reactions, but no nitrite source was provided either from boundary conditions or from other pathways.

In contrast to the central metabolic pathways, the lipid and glycan metabolism had higher variations among different GEMs despite having low median fractions of reactions that were blocked. For example, the glycan metabolism varies from almost fully consistent (STM_v1.0 [72]) to completely blocked (iSB619 [73] and iCce806 [74]). The STM_v1.0 model contained 143 reactions in glycan metabolism. Out of these only five were blocked reactions, which was attributed to a dead-end compound in the “*Murein LPP Biosynthesis*” subsystem. The iSB619 and iCce806, however, contained two and eleven reactions in glycan metabolism, respectively. All of these reactions were blocked and appeared as dead ends of the metabolic models. Similarly, some models (e.g. iND750 [75] and iMM904 [76]) had a large fraction of blocked reactions in lipid metabolism, indicating that the lipid metabolic pathways were incomplete in these models.

The cofactors and vitamins metabolism pathway had slightly higher fractions of blocked reactions (median close to 0.5), and the level of flux inconsistency varies among different GEMs. For example, the model iND750 [75] had a largely blocked cofactors and vitamins metabolism pathway, with 80 of the 104 reactions (77%) unable to produce non-zero fluxes due to the missing connections between metabolites and the biomass function. In contrast, the model iJO1366 [40] had 228 reactions in the cofactors and vitamins metabolism pathway, and only 26 reactions (11%) were blocked.

The secondary and xenobiotics metabolic pathway, while having variable fractions of blocked reactions, only occurred in five GEMs due to their inessentiality to biomass production. Reactions in this pathway can be used to analyze the relationship between biomass production and the metabolism of secondary metabolites. For example, the iCA1273 [77] model has three reactions associated with penicillin breakdown. These reactions are not connected to the biomass production but they are connected appropriately with exchange reactions. Hence, the model can be used to simulate the effect of penicillin on the growth of the organism. Conversely, the four other models (iMM1415 [55], iCce806 [74], RECON2 [78], and RECON2.04) appear to contain a large fraction (0.67 and 0.5, respectively, for iMM1415 and iCce806 and 0.9 for the two RECON models) of blocked reactions in the secondary and xenobiotics metabolism and would require the addition of transport or exchange reactions to simulate functions in this pathway.

It is also worth noting that the total number of reactions was more consistent in the central metabolism than in the pathways of lipid metabolism, glycan metabolism, and transport reactions (Fig 4). The variations among the latter pathways can be attributed to the modeling decisions or the large variability of these pathways among different organisms. For example, some models had experienced an expansion of the glycan and lipid metabolism pathways in later releases compared to the earlier releases. This expansion is apparent when comparing the 2003 *E*. *coli* model iJR904 [79] and the 2007 model iAF1260 [80], and when comparing the 2004 *S*. *cerevisiae* model iND750 [75] with the 2009 model iMM904 [76] (S2 Fig). As the models have evolved through successive updates, a more extensive annotation of glycan and lipid metabolism has been applied, and the lipid and glycan compounds have been included into the biomass function. In contrast to the other pathways, the total number of transport reactions was influenced by the number of cellular compartments in the model. While the GEMs of some eukaryotic organisms contained over 1,000 transport reactions that account for the metabolite exchange among different cellular compartments, GEMs with only two compartments had on average less than 100 transport reactions.

## Discussion

The reconstruction and analysis of GEMs have broad applications in understanding genotype-phenotype connections, studying evolutionary processes, and predicting the outcomes of metabolic engineering [13,81]. Despite the development of tools that support individual steps along the modeling procedure [24,26,29], it is still challenging to associate mathematical simulation with the biological interpretation of GEMs. Often times, model files need to be curated iteratively to set up exchange reactions, assign reaction directionality, define biomass functions, and fill reaction gaps, and the results of mathematical simulations need to be mapped to the annotated data of model definitions. However, due to the absence of integrated data representations and the lack of standardized model checking procedures, the curation of GEMs are prone to introducing inconsistencies, which in turn may lead to errors in modeling and in interpreting the biological meaning of modeling outcomes.

The PSAMM software package was developed to solve the above challenges by integrating the annotation of metabolic pathways with the consistency checking and the constraint-based analyses of mathematical models (Fig 2). It offers a novel YAML format of model representation that integrates the heterogeneous annotation of model components with the formulation of mathematical simulation problems. PSAMM takes advantage of several useful features commonly supported in the Python programming language: (1) it is highly portable and can be easily installed using the pip package management system; (2) it is open source and does not rely on paid software like MATLAB; (3) it has extensive library support that permits both matrix-based operations and dictionary-based feature representations; (4) it is an integrated package that supports calling simulation functions using simple one-line commands (S1 Fig) and permits adjustments to modeling parameters using command options (S1 Table). PSAMM supports the import and export of diverse model formats and is applicable for the curation of draft models generated from model reconstruction pipelines like ModelSEED [26], RAVEN [29], and Pathway Tools [31]. It provides extensive help information (e.g. through the command-line *-h* or --*help* options) and an online tutorial that demonstrates the main functions and workflow of PSAMM (https://psamm.readthedocs.org/en/latest/tutorial.html). Additionally, the PSAMM functionality is easily expandable by the user community through an Application Programming Interface (API).

PSAMM integrates heterogeneous metadata in model annotations with the simulation of metabolic networks, so that users can perform model curation and mathematical simulations under the integrated framework of a unified software package. While COBRA and RAVEN are released as toolboxes and are dependent on the MATLAB software, PSAMM is an independent software package that is freely available for all academic users. Calling the simulation functions is largely simplified in PSAMM because the individual steps of model reading, solver selection, model simulation, and simulation results mapping are integrated into a single one-line command. In contrast, setting up a simulation problem in COBRA would require calling multiple functions in the MATLAB interface (S1 Fig). Moreover, PSAMM allows users to document their modifications to the model definition as well as the simulation conditions during model curations. In contrast, changes made through the MATLAB interface will be lost when the computing session is closed. Even if users write out their changed models into an SBML file, it will be difficult for the users to track the exact location of the changes due to the incompatibility of SBML files with line-based version control systems [51,82]. Finally, PSAMM is configured to perform with high efficiency a number of computationally intensive simulations, such as tFBA, FVA, robustness analysis and random minimal networks (Fig 1 and S2 Table). As an example, we recorded the running time of robustness analysis on iJO1366, which is one of the larger models in the collection we analyzed. Using a single computing thread, the median runtime of robustness analysis with 1000 steps on the EX_o2(e) exchange reaction using Cplex solver was 4.07 seconds in PSAMM (including the reading of model files), whereas COBRA takes 100.7 seconds under the same settings for just solving the simulation problem. This is because PSAMM modifies the simulation problem directly through an optimized LP utility interface (Fig 2), while COBRA requires the simulation problem to be redefined for every step of the robustness analysis.

**Fig 5:**
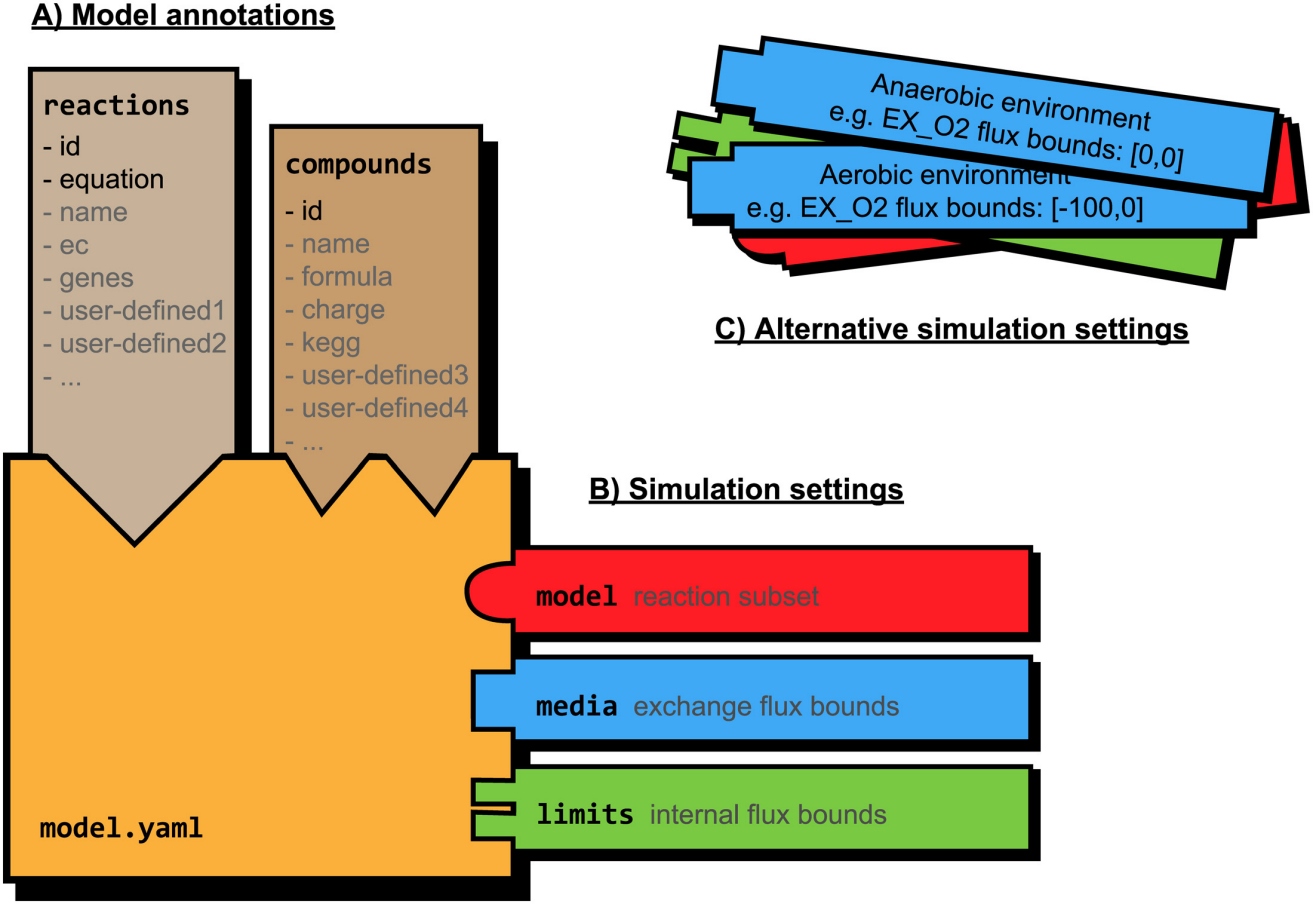
A diagram illustrating the modular representation of model components in the YAML format. The data structure is divided into the static components of model annotation (A) and the dynamic components of simulation settings (B). The reaction and compound annotation databases are associated with a number of required (highlighted in black, e.g. “- id” and “- equation” for reactions) and optional (gray) data entries, and user-defined, model-specific data entries are permitted in the annotation databases. The simulation settings can be represented with various combinations of the model, limits, and media files. Alternative conditions may be defined using a number of alternative modules that can be switched with one another.

The PSAMM YAML-based model representation provides additional support to model curation by offering a human-readable interface for the organization of heterogeneous data (Fig 3 and S1 Text). The YAML format incorporates information about both model annotations (e.g. in the *reactions*, *compounds*, and *model* definitions) and model simulations (e.g. in the *biomass*, *media*, and *limits* definitions), and it supports a flexible interface for storing annotation information either within a centralized model file or as independent feature files. The YAML format streamlines model representation by minimizing the use of markup tags. The use of line breaks and whitespaces as markups of data structure not only enables the parsing and association of user-defined, model-specific model annotations, but also enhances the compatibility of YAML with line-based version control systems. This compatibility with version tracking is especially useful in collaborative projects that involve multiple curators working on the same model since modifications made by different curators can be documented in parallel and later reconciled and merged into a new model release that is more comprehensive than what could be achieved by a single curator. Additionally, YAML supports the modular representation of diverse model components and the free combination of model definitions with diverse simulation conditions (Fig 5). Hence, instead of being restricted to a single simulation condition, YAML provides additional flexibilities for the documentation of multiple modeling conditions in a single model release.

Applying PSAMM to existing GEMs in public literature, a number of inconsistencies were identified. These included inconsistencies in the formatting of model files (e.g. SBML or Excel files), the definition of model stoichiometry, and the presence of blocked reactions. In general, the representation of SBML and Excel files varies among different GEMs both in the formatting of model annotations and in the completeness of information provided. For example, only a small fraction of the collected SBML models included complete metadata regarding protein-coding genes, functional annotations, Enzyme Commission (EC) numbers, and subsystem classifications. This was largely due to the lack of standardized definition of such information in SBML specifications and to some extent has prevented the SBML format from integrating heterogeneous annotation data. The Excel files, although frequently used by model curators for the annotation of pathway information, also lacks standardization and data integration. These limitations have caused a number of common inconsistencies in the representation of both SBML and Excel models. Using the annotation framework of PSAMM and the integrated YAML representation, we have corrected these inconsistencies and documented the changes in an online Git repository at https://github.com/zhanglab/psamm-model-collection (S2–S5 Text).

To ensure that the GEMs correctly represented the function and connectivity of metabolic networks, the PSAMM stoichiometric and flux consistency checking were applied to search for unbalanced or disconnected reactions. Surprisingly, only 30% of the analyzed models were stoichiometrically balanced, and the unbalanced reactions can be attributed to the inconsistent representation of compounds in different reaction equations. The analysis of flux consistency revealed that while the central metabolic pathways contain only a small fraction of blocked reactions across all GEMs, the pathways of lipid metabolism, glycan metabolism, and cofactors and vitamins metabolism were largely variable among different GEMs and were frequently disconnected from biomass production. Interestingly, the pathway distribution of metabolic reactions varied among different GEMs (S2 Fig). In the successive models of certain organisms (e.g. in *E*. *coli*), it appeared that the initial reconstructions have focused on central metabolic pathways, while the later iterations have contributed to the inclusion and completion of peripheral pathways like the transport reactions and the glycan and lipid metabolism. While it is not uncommon to have blocked reactions in GEMs due to the limited knowledge in current literature about certain metabolic pathways, the analysis of flux inconsistency should be a standard step especially in interpreting the biological significance of gene or reaction deletion simulations. Since the blocked reactions do not contribute to the production of biomass, they will always be predicted as non-essential reactions. However, such prediction is not biologically meaningful because it reflects an artifact caused by the disconnection of metabolic pathways.

## Materials and Methods

### Comparisons of Program Running Time

A list of functions (marked in S1 Table) was examined in the PSAMM package as well as the COBRA and RAVEN toolboxes to compare the efficiency of the different tools. The simulations were carried out on the model iJO1366 [40] with the Cplex linear programming solver for PSAMM (version 0.17) and COBRA (version 12.6, IBM academic release for PSAMM and TOMLAB release for COBRA), and with the MOSEK solver for RAVEN (version 7.1.0.36). The specific simulation parameters were set according to the conditions specified for each function in the S2 Table. Outputs from different tools were manually examined to ensure that the same results were generated form the same simulation settings. The running time was recorded in a CentOS operating system with a single processor core allocated to each program (the solver was not restricted in this way and was able to use up to 20 cores). Each simulation was carried out at least seven times, from which the median was plotted in Fig 1 with the 75th and the 25th percentile values as the upper and the lower limits of the error bar.

### Dependencies

PSAMM has the following dependencies, some of which are required and some of which are optional but limit the functionality of PSAMM in their absence. The dependencies PyYAML, xlrd, xlsxwriter, and NumPy can be automatically installed through the Python package manager pip. The linear programming solvers need to be installed by the user following instructions from corresponding releases.

- PyYAML (required, for parsing the PSAMM YAML format)
- IBM ILOG Cplex Optimizer (required, for solving linear programming problems)
- GUROBI Optimizer (optional, an alternative to the Cplex linear programming solver)
- QSopt_ex rational solver [83] (optional, for obtaining exact solutions to the LP problem)
- xlrd (optional; for reading models from Excel sheet files)
- xlsxwriter (optional; for exporting YAML models as Excel sheet files)
- NumPy (optional; for converting the sparse matrix representation to the NumPy matrix)

### Collection of Metabolic Models

A list of 56 GEMs was collected from current literature and a public model collection [84] as SBML files. Additionally, the RECON2.04 model was downloaded in the format of a MATLAB data file from a public release at https://vmh.uni.lu/#downloadview. Compound and reaction features of nine models were downloaded in the Microsoft Excel format (S3 Table). These models were used to test the application of PSAMM for model management and model curation. For constraint-based simulations, a biomass reaction was identified for each model by examining the objective coefficient defined under the SBML “kineticLaw” section. The reaction with a non-zero objective coefficient was treated as the biomass reaction. If the objective function was not specified in SBML file, the reaction identifiers were searched and the ones containing the string “biomass” were marked as biomass reactions. Information was also obtained from original publications of the collected models to identify biomass reactions and to select the main biomass reaction when more than one was present. The biomass reactions of each model were listed in S3 Table.

### Stoichiometric Consistency Checking

The stoichiometric consistency check was implemented in the PSAMM *masscheck* function, which supports both compound-based and reaction-based checking of the stoichiometric consistency. By default, the *masscheck* function excludes the exchange and biomass reactions from consideration because by design, stoichiometric balance is not required in these reactions. The compound-based stoichiometric consistency checking was implemented based on Thiele et al. [49] and was formulated as:

Maximize *Σ*_*i*_ *z*_*i*_

Subject to

- ***S*** ^*T*^ ***m*** = 0
- For all *m*_*i*_ in ***m*:** *m*_*i*_≥*z*_*i*_
- For all *z*_*i*_ in ***z*:** *z* ∈[0;1]

This problem looks for compounds that failed to obtain a positive mass under optimizing conditions. By maximizing the values in ***z*** up to 1 for as many compounds as possible, it forced the mass values (***m***) to be at least 1 for the same compounds. The sum of the values in ***z*** can be used to approximate the number of compounds that were properly balanced.

Although the compound-based checking was useful for identifying compounds that may cause stoichiometric inconsistency, it provides no insights into the stoichiometric balance of individual reactions. Therefore, PSAMM implements a new algorithm to directly search for unbalanced reactions. This approach is performed using an LP problem that was modified from the above stoichiometric consistency check. A mass residual variable (***r***) is introduced into every reaction and bounded by the residual bound variable (***z***) such that *r*_*j*_ ∈[−*z*_*j*_ *;z*_*j*_]. The residual bound variables are then minimized, and the reactions that carry a non-zero residual value *r*_*j*_ (i.e. bounded by a positive *z*_*j*_) are reported as a candidate of inconsistency. The mathematical formulation of this approach is described in the following LP problem:

Maximize *Σ*_*j*_ *z*_*j*_

Subject to

- ***S*** ^*T*^ ***m*** = ***r***
- For all *m*_*i*_ in ***m*:** *m*_*i*_≥1
- For all *z*_*j*_ in ***z*:** *z*_*j*_≥0
- For all *r*_*i*_ in ***r*:** *r*_*j*_ ∈[−*z*_*j*_ *;z*_*j*_]

This approach can be applied iteratively to assist with the correction of stoichiometric inconsistencies in metabolic models. The residual variables indicate a mass that is missing from identified reactions, and the sign of the residual can be used to determine whether the left or right side of an equation has a missing mass. Additionally, PSAMM provides a “--*checked*” option in the *masscheck* function, which allows fixing certain residual values at zero when the corresponding reactions have been confirmed to be balanced, making the program able to converge on the remaining set of unbalanced reactions. When the stoichiometric consistency check was performed on the collection of models, the biomass and exchange reactions were excluded from consideration and the requirement of a positive mass was removed from compounds that were used to model photons or electrons.

### Flux Consistency Checking

The flux consistency check was implemented in the PSAMM *fluxcheck* function. A *flux inconsistent reaction* is a reaction that cannot take a non-zero flux under any simulation conditions. In other words, given the stoichiometric matrix ***S***, the vector of fluxes ***v***, and the reaction *j*, if no solution to ***Sv*** *=* ***0*** exists, where *v*_*j*_ ≠ 0 the reaction is considered to be flux inconsistent. Conversely, if a solution exists the reaction is considered to be flux consistent. A consistent model is a model that only contains consistent reactions [85]. The flux consistency check in PSAMM was implemented based on two independent approaches. The first approach was based on the definitions in [86]. This was achieved by applying a modified version of the flux variability analysis (FVA) on each reaction while using default constraints on other reactions. For reversible reactions, the flux is first maximized and then minimized, and for irreversible reactions, only the maximization is necessary. A significant speedup was possible in PSAMM (S2 Table) by avoiding regenerating new problem definitions throughout the procedure. In previous implementations (e.g. COBRA) the LP problem was regenerated for optimizing each reaction. However, in PSAMM the LP problem definition was instead reused. This was feasible since the LP problems in this approach are all equivalent except for using a different objective function for each reaction. Reactions that carried non-zero fluxes were considered to be flux consistent, and vice versa. Alternatively, PSAMM provides another way of checking reaction flux consistency based on the fast consistency check (FASTCC) algorithm, which was designed to provide a more efficient solution in evaluating reaction flux consistency [87]. When analyzing the models in the collection for flux inconsistencies, the constraints on the flux limits of the exchange reactions were removed prior to the analysis to eliminate the influences of external settings and to provide a lower estimate for the fraction of inconsistent reactions (Fig 4).

## Supporting Information

S1 Fig Comparison of the user interfaces in PSAMM versus COBRA. While PSAMM operates as a command-line tool, COBRA is a toolbox dependent on the MATLAB environment. The red font indicates commands typed in by users, and the black font indicates outputs from running a given command.
(TIF)

S2 Fig Distribution of model reactions by metabolic pathways in 19 models. The RECON2 and RECON2.04 were plotted in a different panel, with iMM1415 in both panels as a reference to show the differences in scaling y-axis.
(TIF)

S1 Table Comparison of the common constraint-based modeling functions in PSAMM, RAVEN, and COBRA. The marked rows in the column "Time" indicate commands for which the running time was recorded.
(PDF)

S2 Table Comparisons of the time needed to use various functions in PSAMM, COBRA, and RAVEN. The function is listed in the first column along with information on the parameters used in the simulation. The specific commands, running times for seven independent experiments, along with the 25 and 75 percentile times and the median time taken to run the commands are listed in the remaining columns.
(PDF)

S3 Table List of metabolic models analyzed. The table shows the model identifiers (column 1); the scientific names of the modeled organisms (column 2); the domain of life that each organism belongs to (column 3); the journal, authors and year of the model publications (columns 4–6); whether the Excel format of a model was analyzed (column 7); the SBML level used to format each model’s SBML release (column 8); whether the subsystem information was available for model comparison (column 9); the main biomass reaction used to simulate model growth (column 10); additional biomass reactions included in the model (column 11); whether the model is stoichiometrically consistent (column 12); and the percentage of internal reactions in the model that were blocked (column 13).
(PDF)

S4 Table Mapping of model subsystems to metabolic pathways. The model-specific subsystem names in the first column are mapped to the metabolic pathways in the second column.
(PDF)

S1 Text Description of the PSAMM YAML model format. (PDF)

S2 Text Comparison of the original version and the fixed version of iJN746 [58] using the *git diff* function in the Git version control system. The original version cannot provide a non-zero flux in FBA simulations because the periplasmic cardiolipin compounds (M_clpn120_p, M_clpn160_p, M_clpn161_p, M_clpn180_p, M_clpn181_p) were mislabeled as cytosolic compounds in the SBML model. The updated model correctly incorporates the mislabeled compounds and is able to produce a non-zero flux on biomass. The lines are color coded to highlight manual corrections made to the model. Highlighted in red are the lines removed, and highlighted in green are the lines added from the original version to the new version.
(PDF)

S3 Text Comparison of the original version and the fixed version of the model iRsp1095 [66] model using the *git diff* function in the Git version control system. The original version cannot provide a non-zero flux in FBA simulations, while the fixed version can. This was determined to be due to all of the exchange reactions being set to have a lower bound of zero thus preventing uptake of any nutrients. The exchange reactions were altered to reflect the boundary conditions stated in the supplemental materials of the original paper [66]. The lines are color coded to highlight the changes between the two versions. Highlighted in red are the lines removed, and highlighted in green are the lines added from the original version to the new version.
(PDF)

S4 Text Comparison of the original version and the fixed version of the model iMA871 [67] model using the *git diff* function in the Git version control system. The original version cannot provide a non-zero flux in FBA simulations, while the fixed version can. The problem was determined to be that there was no sink for the artificial biomass compound, *M_BIOMASS*, meaning it could accumulate but could not be counted towards biomass production. A sink was added for the compound allowing the model to generate a non-zero biomass flux. The lines are color coded to highlight the changes between the two versions. Highlighted in red are the lines removed, and highlighted in green are the lines added from the original version to the new version.
(PDF)

S5 Text Comparison of the original version and the updated version (downloaded on September 09, 2015 from http://www.maranasgroup.com/models.htm) of the model iSyn731 [65] using the *git diff* function in the Git version control system. The original version cannot provide a non-zero flux in FBA simulations. The new version of the model contained several changes including reaction directionality changes, stoichiometric changes, and the addition and deletion of reactions. The lines are color coded to highlight the changes between the two versions. Highlighted in red are the lines removed, and highlighted in green are the lines added from the original version to the new version.
(PDF)
