# Supplementary figures and images for: PSAMM: A Portable System for the Analysis of Metabolic Models
Source: PLoS Comput Biol. 2016 Feb 1;12(2):e1004732. doi: 10.1371/journal.pcbi.1004732 (PMC4734835; doi:10.1371/journal.pcbi.1004732)

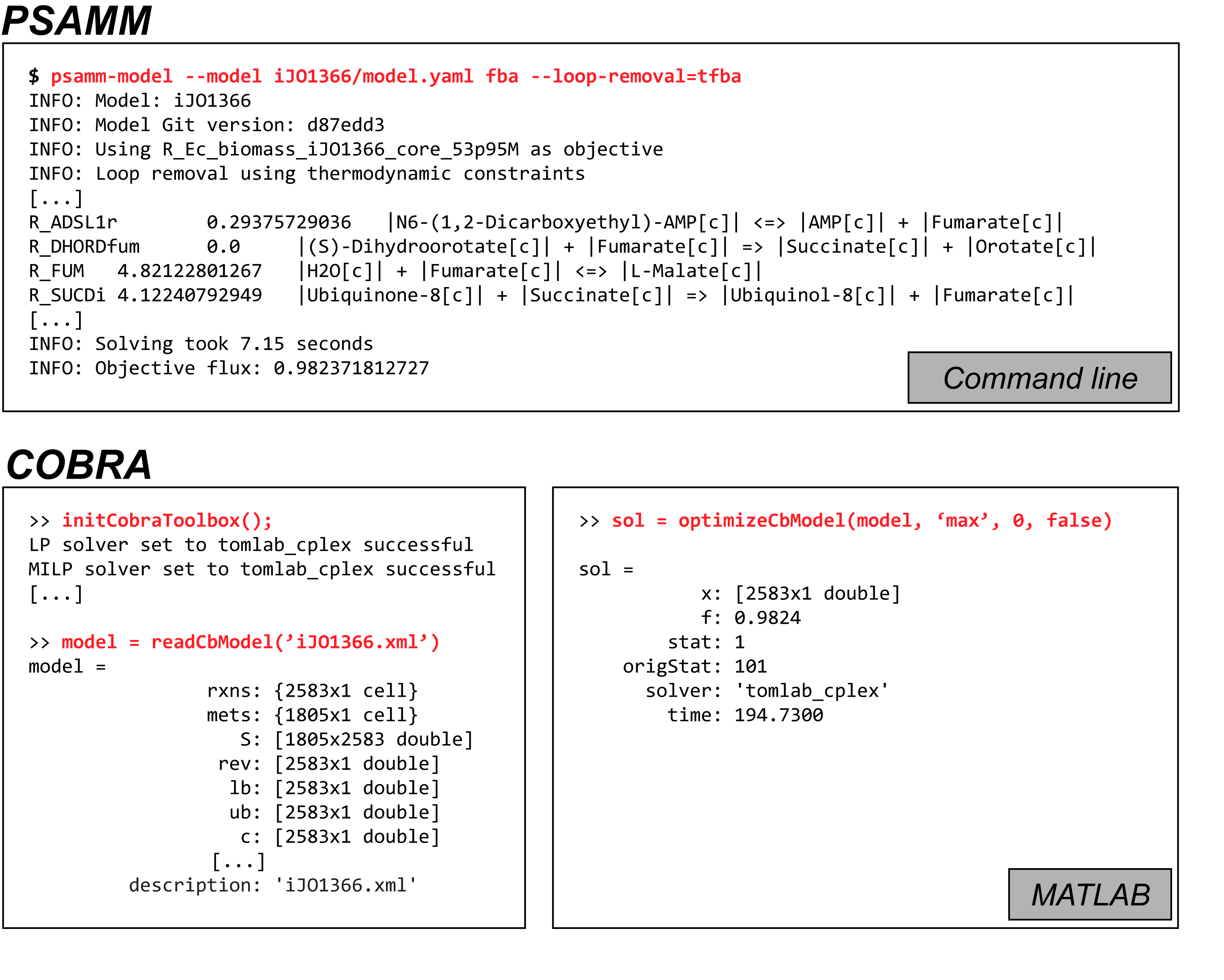

Supplement: S1 Fig — While PSAMM operates as a command-line tool, COBRA is a toolbox dependent on the MATLAB environment. The red font indicates commands typed in by users, and the black font indicates outputs from running a given command. (TIF) [file pcbi.1004732.s001.tif]

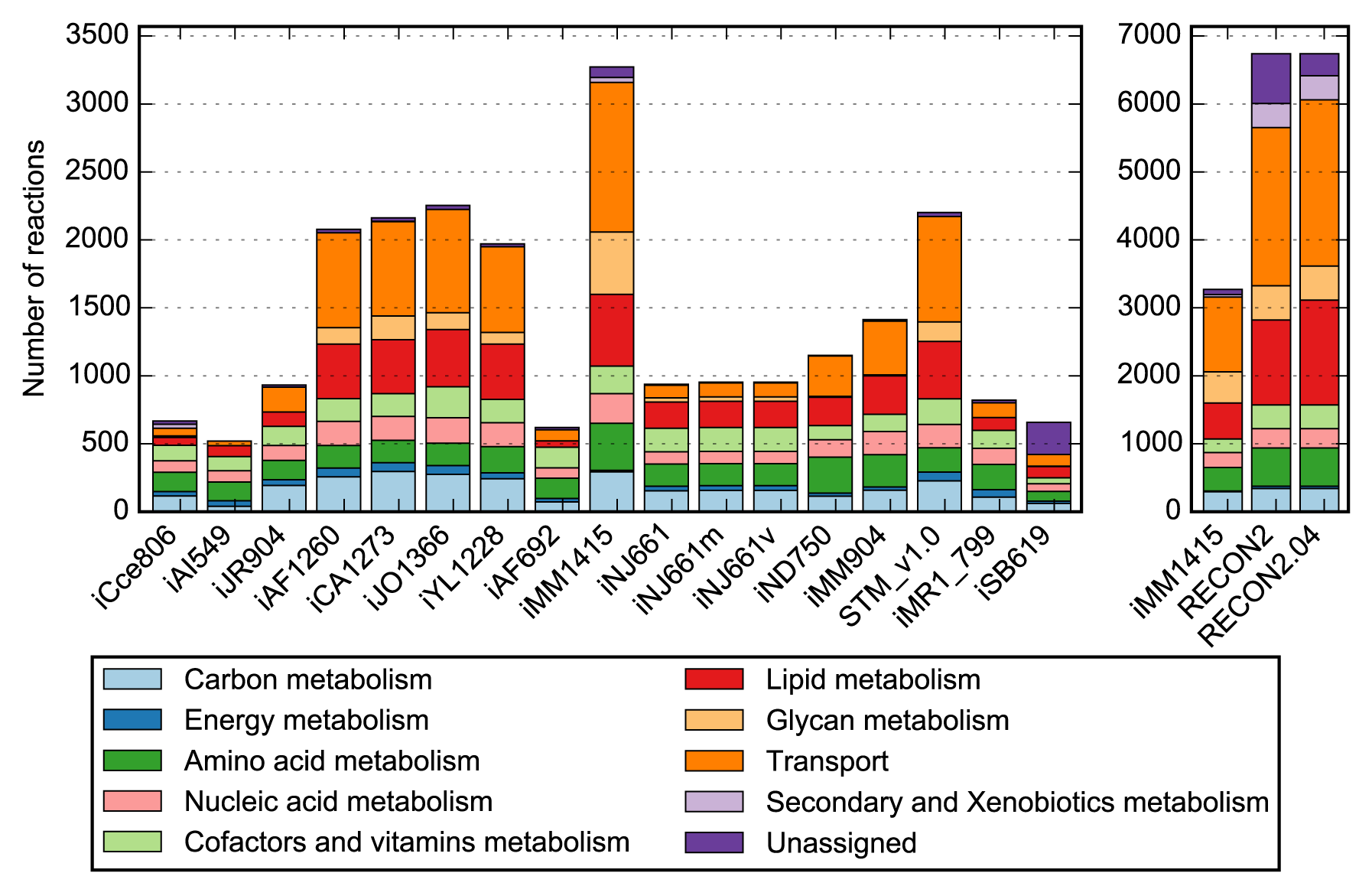

Supplement: S2 Fig — The RECON2 and RECON2.04 were plotted in a different panel, with iMM1415 in both panels as a reference to show the differences in scaling y-axis. (TIF) [file pcbi.1004732.s002.tif]
