# Supplementary material for: PSAMM: A Portable System for the Analysis of Metabolic Models
Source: PLoS Comput Biol. 2016 Feb 1;12(2):e1004732. doi: 10.1371/journal.pcbi.1004732 (PMC4734835; doi:10.1371/journal.pcbi.1004732)
Supplement: S1 Table — The marked rows in the column "Time" indicate commands for which the running time was recorded. (PDF) [file pcbi.1004732.s003.pdf]

S1 Table. Comparison of the common constraint-based modeling functions in PSAMM, RAVEN, and COBRA. The marked rows in the column "Time" indicates commands for which the running time was recorded.

Type of Functions	Functions	Time	PSAMM		RAVEN		COBRA	
			Included	Command	Included	Command	included	function
Import/export	import Excel		Yes	psamm-import <Model_name>[a]	Yes	importExcelModel	Yes	xls2model
	import SBML	X	Yes	psamm-import sbml, psamm-import sbml-strict	Yes	importModel	Yes	readCbModel
	import JSON		Yes	psamm-import json	No	-	No	-
	import ModelSEED model		Yes	psamm-import modelseed	No	-	No	-
	import from KEGG model		No	-	Yes	getKEGGModelForOrganism	No	-
	export to Excel		Yes	psamm-model excelexport	Yes	exportToExcelFormat	Yes	writeCbModel
	export to SBML		Yes	psamm-model sbmlexport	Yes	SBMLFromExcel	Yes	writeCbToSBML
	export YAML		Yes	psamm-import	No	-	No	-
Model checking	Charge Balance	X	Yes	psamm-model chargecheck	No	-	Yes	checkMassChargeBalance
	Formula Balance	X	Yes	psamm-model formulacheck	Yes	getElementalBalance	Yes	checkMassChargeBalance
	Stoichiometric Checking (identifies unbalanced compouds)	X	Yes	psamm-model masscheck	No	-	Yes	checkStoichiometricConsistency
	Stoichiometric Checking (identifies unbalanced reactions)		Yes	psamm-model masscheck --type=reaction	No	-	No	-
	Flux Consistency (FastCC algorithm)	X	Yes	psamm-model fluxcheck --fastcore	No	-	Yes	fastcc
	Flux Consistency (zero-flux reactions, FVA)	X	Yes	psamm-model fluxcheck	No	-	Yes	findBlockedReaction
	Flux Consistency (zero-flux reactions, tFVA)[b]		Yes	psamm-model fluxcheck --tfva	No	-	No	-
	GapFill		Yes	psamm-model gapfill	Yes	gapReport, fillGaps	No[d]	-
	FastGapFill		Yes	psamm-model fastgapfill	No		Yes	fastGapFill
Constraint Based Analysis	Flux Balance Analysis	X	Yes	psamm-model fba	Yes	solveLP	Yes	optimizeCbModel
	FBA with thermodynamic constraints	X	Yes	psamm-model fba --loop-removal=tfba	No	-	Yes[e]	addLoopLawConstraints, optimizeCbModel
	Flux Variability Analysis	X	Yes	psamm-model fva	No[c]	getAllowedBounds	Yes	fluxVariability
	Robustness Analysis	X	Yes	psamm-model robustness	No	-	Yes	robustnessAnalysis
	Robustness Analysis w/ thermodynamic constraints		Yes	psamm-model robustness --loop-removal=tfba	No	-	No	-
	Random Minimal Network Analysis	X	Yes	psamm-model randomsparse	No	-	No	-

[a]. The Excel import functions were developed for individual models because there is no consensus on the representation of GEMs in Excel format.
[b]. tFVA is the flux variable analysis with thermodynamic constraints.
[c]. The getAllowedBounds function works very similar to FVA but does not impose a constraint on the biomass reaction.
[d]. COBRA only releases a function for GapFind, but not GapFill.
[e]. This function works with the commercial release of the Cplex solver from TOMLAB, but it does not appear to work with cplex_direct (the academic release) or by solving the MPS file in the Cplex command line interface.
