# Supplementary material for: PSAMM: A Portable System for the Analysis of Metabolic Models
Source: PLoS Comput Biol. 2016 Feb 1;12(2):e1004732. doi: 10.1371/journal.pcbi.1004732 (PMC4734835; doi:10.1371/journal.pcbi.1004732)
Supplement: S2 Table — The function is listed in the first column along with information on the parameters used in the simulation. The specific commands, running times for seven independent experiments, along with the 25 and 75 percentile times and the median time taken to run the commands are listed in the remaining columns. (PDF) [file pcbi.1004732.s004.pdf]

S2 Table: Comparsions of the time needed to use various functions in PSAMM, COBRA, and RAVEN. The function is listed in the first column along with information on the parameters used in the simulation. The specific commands, running times for seven independent experiments, along with the 25th and 75th percentile times and the median time taken to run the commands are listed in the remaining columns.

	Experiment Number	PSAMM (Cplex 12.6) (**including time to load model)**	PSAMM (Cplex 12.6) (only solving)	COBRA (TOMLAB Cplex, Cplex 12.6)	RAVEN (MOSEK 7.1.0.36)
**Load SBML model**		psamm-import sbml --source iJO1366.xml		model = readCbModel('iJO1366.xml')	model = importModel('iJO1366.xml', true, true)
	1	5.92	5.92	17.14	4.45
	2	5.76	5.76	17.22	3.93
iJO1366, load from SBML file to memory representation. For PSAMM, converting to YAML format.	3	5.83	5.83	17.24	3.93
	4	5.95	5.95	17.37	3.95
	5	5.85	5.85	17.25	3.93
	6	5.92	5.92	17.06	3.77
	7	5.90	5.90	17.25	3.90
	25% percentile	5.84	5.84	17.18	3.92
	Median	5.90	5.90	17.24	3.93
	75% percentile	5.92	5.92	17.25	3.93
**Standard FBA**		psamm-model fba		sol = optimizeCbModel(model)	sol = solveLP(model)
	1	3.88	0.03	0.08	0.32
	2	3.10	0.03	0.07	0.29
iJO1366, no thermodynamic constraints. Solver can use up to 20 threads.	3	3.16	0.03	0.13	0.29
	4	3.13	0.03	0.11	0.29
	5	3.08	0.03	0.07	0.30
	6	3.13	0.03	0.09	0.29
	7	3.07	0.03	0.07	0.30
	25% percentile	3.09	0.03	0.07	0.29
	Median	3.13	0.03	0.08	0.29
	75% percentile	3.14	0.03	0.10	0.30
**FBA with thermodynamic constraints**		psamm-model fba --loop-removal=tfba		sol = optimizeCbModel(model, 'max', 0, false)	
	1	10.88	7.07	195.1	N/A
	2	10.13	7.20	195.4	N/A
iJO1366, with thermodynamic constraints. Solver can use up to 20 threads.	3	9.96	7.09	193.7	N/A
	4	9.80	6.99	194.7	N/A
	5	9.80	7.01	196.2	N/A
	6	9.78	6.92	191.8	N/A
	7	9.80	6.91	193.4	N/A
	25% percentile	9.80	6.96	193.55	N/A
	Median	9.80	7.01	194.7	N/A
	75% percentile	10.05	7.08	195.25	N/A
**Robustness**		psamm-model robustness --steps 1000 R_EX_o2_LPAREN_e_RPAREN_		[controlFlux, objFlux] = robustnessAnalysis(model, 'EX_o2(e)', 1000)	
	1	4.89	1.38	94.8	N/A
iJO1366, EX_o2(e), no thermodynamic constraints, 1000 steps. The number of parallel pool workers was set to one. Solver can use up to 20 threads.	2	4.05	1.38	100.7	N/A
	3	4.07	1.38	96.8	N/A
	4	4.04	1.38	100.8	N/A
	5	4.07	1.38	100.5	N/A
	6	4.19	1.38	101.2	N/A
	7	4.05	1.38	102.8	N/A
	25% percentile	4.05	1.38	98.65	N/A
	Median	4.07	1.38	100.7	N/A
	75% percentile	4.13	1.38	101.00	N/A
**FVA**		psamm-model fva		[minFlux, maxFlux] = fluxVariability(model)	
	1	9.12	7.03	75.3	N/A
	2	8.85	7.06	75.3	N/A
iJO1366, no thermodynamic constraints. The number of parallel pool workers was set to one. Solver can use up to 20 threads.	3	8.86	7.03	75.2	N/A
	4	8.26	7.01	75.1	N/A
	5	8.31	7.05	75.1	N/A
	6	8.27	7.02	75.4	N/A
	7	8.30	7.03	75.6	N/A
	25% percentile	8.29	7.03	75.15	N/A
	Median	8.31	7.03	75.3	N/A
	75% percentile	8.86	7.04	75.35	N/A
**Random minimal network (randomsparse)**		psamm-model randomsparse --type genes 30%			
	1	1662	N/A	N/A	N/A
	2	1623	N/A	N/A	N/A
iJO1366, gene-based, no thermodynamic constraints, 30% threshold. 100 random solutions.	3	1817	N/A	N/A	N/A
	4	1670	N/A	N/A	N/A
	5	1653	N/A	N/A	N/A
	6	1664	N/A	N/A	N/A
	7	1674	N/A	N/A	N/A
	25% percentile	1657.5	N/A	N/A	N/A
	Median	1664.0	N/A	N/A	N/A
	75% percentile	1672.0	N/A	N/A	N/A
**Random minimal network (randomsparse)**		psamm-model randomsparse --type reactions 30%			
	1	3502	N/A	N/A	N/A
	2	3512	N/A	N/A	N/A
iJO1366, reaction-based, no thermodynamic constraints, 30% threshold. 100 random solutions.	3	3524	N/A	N/A	N/A
	4	3538	N/A	N/A	N/A
	5	3698	N/A	N/A	N/A
	6	3486	N/A	N/A	N/A
	7	3534	N/A	N/A	N/A
	25% percentile	3507.0	N/A	N/A	N/A
	Median	3524.0	N/A	N/A	N/A
	75% percentile	3536.0	N/A	N/A	N/A

**Formula balance check**		psamm-model formulacheck		model = findSExRxnInd(model)<br>model.SIntRxnBool(find(strcmp(model.rxns, 'Ec_biomass_iJO1366_WT_53p95M'))) = 0<br>[massImbalance, imBalancedMass, imBalancedCharge, imBalancedRxnBool, Elements, missingFormulaeBool, balancedMetBool] = checkMassChargeBalance(model, 2)	balance = getElementalBalance(model)
	1	2.59	N/A	13.9	2.8
	2	2.32	N/A	13.8	2.7
iJO1366. COBRA combines formula check and charge check in one function. The time for COBRA is the time to run that function.	3	2.33	N/A	13.8	2.7
	4	2.40	N/A	13.7	2.7
	5	2.35	N/A	13.8	2.7
	6	2.44	N/A	14.0	2.7
	7	2.42	N/A	13.8	2.7
	25% percentile	2.34	N/A	13.80	2.70
	Median	2.40	N/A	13.80	2.70
	75% percentile	2.43	N/A	13.85	2.70
**Charge balance check**		psamm-model chargecheck		See above	
	1	1.67	N/A		N/A
	2	1.55	N/A		N/A
	3	1.62	N/A		N/A
iJO1366.	4	1.63	N/A		N/A
	5	1.63	N/A		N/A
	6	1.61	N/A		N/A
	7	1.67	N/A		N/A
	25% percentile	1.61	N/A		N/A
	Median	1.63	N/A		N/A
	75% percentile	1.65	N/A		N/A
**Stoichiometric balance check**		psamm-model masscheck		model = findSExRxnInd(model)<br>model.SIntRxnBool(find(strcmp(model.rxns, 'Ec_biomass_iJO1366_WT_53p95M'))) = 0<br>[inform, m] = checkStoichiometricConsistency(model)	
	1	3.13	1.09	0.1	N/A
	2	3.10	1.05	0.1	N/A
	3	3.07	1.03	0.1	N/A
iJO1366. Only compound imbalance check.	4	3.15	1.16	0.1	N/A
	5	2.95	0.91	0.1	N/A
	6	2.86	0.83	0.1	N/A
	7	3.17	1.07	0.1	N/A
	25% percentile	3.01	0.97	0.09	N/A
	Median	3.10	1.05	0.1	N/A
	75% percentile	3.14	1.08	0.09	N/A
**Flux consistency check (zero-flux reactions)**		psamm-model fluxcheck		BlockedReaction = findBlockedReaction(model)	
	1	12.62	N/A	79.6	N/A
	2	12.02	N/A	79.5	N/A
iJO1366. Using FVA (without biomass constraint). The number of parallel pool workers was set to one. Solver can use up to 20 threads.	3	11.98	N/A	79.0	N/A
	4	11.88	N/A	78.5	N/A
	5	11.91	N/A	78.5	N/A
	6	11.90	N/A	78.5	N/A
	7	11.95	N/A	78.4	N/A
	25% percentile	11.91	N/A	78.50	N/A
	Median	11.95	N/A	78.5	N/A
	75% percentile	12.00	N/A	79.25	N/A
**Flux consistency check (thermodynamic constraints)**		psamm-model fluxcheck --tfba			
	1	7619	N/A	N/A	N/A
	2	7648	N/A	N/A	N/A
	3	7623	N/A	N/A	N/A
iJO1366.	4	7608	N/A	N/A	N/A
	5	7596	N/A	N/A	N/A
	6	7637	N/A	N/A	N/A
	7	7606	N/A	N/A	N/A
	25% percentile	7607.0	N/A	N/A	N/A
	Median	7619	N/A	N/A	N/A
	75% percentile	7630.0	N/A	N/A	N/A
**Flux consistency check (fastcc)**		psamm-model fluxcheck --fastcore		[A, V] = fastcc(model, 1e-5, 1)	
	1	14.52	N/A	22.0	N/A
	2	14.52	N/A	21.7	N/A
	3	14.62	N/A	21.8	N/A
iJO1366.	4	14.57	N/A	22.5	N/A
	5	14.50	N/A	21.6	N/A
	6	14.52	N/A	21.5	N/A
	7	14.68	N/A	21.6	N/A
	25% percentile	14.52	N/A	21.60	N/A
	Median	14.52	N/A	21.7	N/A
	75% percentile	14.60	N/A	21.90	N/A
