# Supplementary material for: PSAMM: A Portable System for the Analysis of Metabolic Models
Source: PLoS Comput Biol. 2016 Feb 1;12(2):e1004732. doi: 10.1371/journal.pcbi.1004732 (PMC4734835; doi:10.1371/journal.pcbi.1004732)
Supplement: S3 Table — The table shows the model identifiers (column 1); the scientific names of the modeled organisms (column 2); the domain of life that each organism belongs to (column 3); the journal, authors and year of the model publications (columns 4–6); whether the Excel format of a model was analyzed (column 7); the SBML level used to format each model’s SBML release (column 8); whether the subsystem information was available for model comparison (column 9); the main biomass reaction used to simulate model growth (column 10); additional biomass reactions included in the model (column 11); whether the model is stoichiometrically consistent (column 12); and the percentage of internal reactions in the model that were blocked (column 13). (PDF) [file pcbi.1004732.s005.pdf]

S3 Table. List of metabolic models analyzed. The table shows the model identifiers (column 1); the scientific names of the modeled organisms (column 2); the domain of life that each organism belongs to (column 3); the journal, authors and year of the model publications (columns 4-6); whether the Excel format of a model was analyzed (column 7); the SBML level used to format each model's SBML release (column 8); whether the subsystem information was available for model comparison (column 9); the main biomass reaction used to simulate model growth (column 10); additional biomass reactions included in the model (column 11); whether the model is stoichiometrically consistent (column 12); and the percentage of internal reactions in the model that were blocked (column 13).

Model identifier	Organism	Domain	Reference	Journal	Date	Excel Format	Matlab Format	SBML level	Subsystem Analyzed	Biomass reaction	Alternative biomass reactions	Stoichiometric Consistency	% Blocked Reactions
iFF708	*Saccharomyces cerevisiae*	Eukaryota	Forster et al.	Genome Res	2003	No	No	2	No	R_VGRO		No	27.20%
iJR904	*Escherichia coli*	Bacteria	Reed et al.	Genome Biol	2003	No	No	2	Yes	R_BiomassEcoli		Yes	17.70%
iND750	*Saccharomyces cerevisiae*	Eukaryota	Duarte et al.	Genome Res	2004	No	No	2	Yes	R_biomass_SC4_bal		Yes	43.91%
iIB711	*Streptomyces coelicolor*	Bacteria	Borodina et al.	Genome Res.	2005	No	No	2	No	R_r0793		No	6.88%
iIT341	*Helicobacter pylori*	Bacteria	Thiele et al.	J Bacteriol	2005	No	No	2	No	R_BiomassHP_published		Yes	9.64%
iSB619	*Staphylococcus aureus*	Bacteria	Becker et al.	BMC Microbiol	2005	No	No	2	Yes	R_biomass_SA_8a	R_SA_biomass_1a, R_biomass_SA_2a, R_biomass_SA_2b, R_biomass_SA_3a, R_biomass_SA_3b, R_biomass_SA_4a, R_biomass_SA_5a, R_biomass_SA_6a, R_biomass_SA_6b, R_biomass_SA_7a, R_biomass_SA_7b, R_biomass_SA_lipids_only, R_biomass_SA_nuc_only, R_biomass_SA_only_AA	Yes	24.35%
iAF692	*Methanosarcina barkeri*	Archaea	Feist et al.	Mol Syst Biol	2006	No	No	2	Yes	R_Mb_biomass_30		Yes	20.97%
iAF1260	*Escherichia coli*	Bacteria	Feist et al.	Mol Syst Biol	2007	No	No	2	Yes	R_Ec_biomass_iAF1260_core_59p81M		Yes	9.82%
iNJ661	*Mycobacterium tuberculosis*	Bacteria	Jamshidi et al.	BMC Syst Biol	2007	Yes	No	2	Yes	R_biomass_Mtb_9_60atp		No	22.39%
RECON 1 (Downloaded from BiGG Database)	*Homo sapiens*	Eukaryota	Duarte et al.	PNAS	2007	No	No	3	No	N/A		No	35.76%
mus_musculus	*Mus musculus*	Eukaryota	Quekn et al.	Genome Inform.	2008	No	No	2	No	BIO028		No	58.87%
AORYZAE_COBRA	*Aspergillus oryzae*	Eukaryota	Vongsangnak et al.	BMC Genomics	2008	No	No	2	No	R_r2359	R_r1897, R_r1898	No	35.06%
iAC560	*Leishmania major*	Eukaryota	Chavali et al.	Mol Syst Biol	2008	No	No	2	No	R_BIOMASS_LM3		No	36.93%
iJN746	*Pseudomonas putida*	Bacteria	Nogales et al.	BMC Syst Biol	2008	No	No	2	No	R_BiomassKT_TEMP		Yes	14.23%
iJP815	*Pseudomonas putida*	Bacteria	Puchalka et al.	PLoS Comput Biol	2008	No	No	2	No	EX_EC9324		No	35.30%
iMA871	*Aspergillus niger*	Eukaryota	Andersen et al.	Mol Syst Biol	2008	No	No	2	No	R_1024		No	13.88%
iMO1056	*Pseudomonas aeruginosa*	Bacteria	Oberhardt et al.	J Bacteriol	2008	No	No	3	No	R_PA_Biomass6_DM	R_DM_EC_PREGRO	No	23.79%
iBsu1103	*Bacillus subtilis*	Bacteria	Henry et al.	Genome Biol	2009	No	No	2	No	bio00006		Yes	19.42%
iMA945	*Salmonella enterica*	Bacteria	AbuOun et al.	J Biol Chem	2009	Yes	No	2	No	R_ST_biomass_core		No	31.99%
iMM904	*Saccharomyces cerevisiae*	Eukaryota	Mo et al.	BMC Syst Biol	2009	No	No	2	Yes	R_biomass_SC5_notrace		Yes	38.08%
iPS189_fixed	*Mycoplasma genitalium*	Bacteria	Suthers et al.	PLoS Comput Biol	2009	No	No	2	No	R_Biomass		Yes	22.73%
iTZ479	*Thermotoga maritima*	Bacteria	Zhang et al.	Science	2009	No	No	2	No	R_BiomassEcoli_TM		Yes	17.41%
AbyMBEL891	*Acinetobacter baumannii*	Bacteria	Kim et al.	Mol Biosyst	2010	No	No	1	No	R761		No	37.96%
AraGEM	*Arabidopsis thaliana*	Eukaryota	de Oliveira Dal'Molin et al.	Plant Physiol	2010	No	No	2	No	R_BIO_L		No	58.88%
iAI549	*Dehalococcoides ethenogenes*	Bacteria	Ahsanul et al.	PLoS Comput Biol	2010	No	No	2	Yes	R_BIO_CBDB1_DM_855		Yes	40.00%
iMM1415	*Mus musculus*	Eukaryota	Sigurdsson et al.	BMC Syst Biol	2010	No	No	3	Yes	R_biomass_mm_1_no_glygln		No	21.67%
iNJ661m	*Mycobacterium tuberculosis*	Bacteria	Fang et al.	BMC Syst Biol	2010	Yes	No	2	Yes	R_biomass_Mtb_9_60atp_test_NOF		No	18.47%
iNJ661v	*Mycobacterium tuberculosis*	Bacteria	Fang et al.	BMC Syst Biol	2010	Yes	No	2	Yes	R_biomass_Mtb_9_60atp_test_NOF		No	19.94%
iSR432	*Clostridium thermocellum*	Bacteria	Roberts et al.	BMC Syst Biol	2010	No	No	2	No	R_biomass_target		No	40.54%
iSyn669	*Synechocystis sp. PCC6803*	Bacteria	Montagud et al.	BMC Syst Biol	2010	No	No	2	No	R_Biomass		No	43.29%
iTH366	*Plasmodium falciparum*	Eukaryota	Plata et al.	Molecular Systems Biology	2010	No	No	2	No	R_biomass		No	35.02%
PpaMBEL1254	*Pichia pastoris*	Eukaryota	Sohn et al.	Biotechnol J	2010	No	No	2	No	R01288		No	43.33%
PpuMBEL1071	*Pseudomonas putida*	Bacteria	Sohn et al.	Biotechnol J	2010	No	No	2	No	R_Biomass		No	38.19%
S_coelicolor_fixed	*Streptomyces coelicolor*	Bacteria	Alam et al.	BMC Genomics	2010	No	No	2	No	R_biomass		No	16.24%
iCA1273	*Escherichia coli*	Bacteria	Archer et al.	BMC Genomics	2011	No	No	2	Yes	R_Ec_biomass_iCA1273_core_59p81M		No	10.74%
iCB925	*Clostridium beijerinckii*	Bacteria	Milne et al.	BMC Syst Biol	2011	No	No	2	No	R_biomass		Yes	47.23%
iJO1366	*Escherichia coli*	Bacteria	Orth et al.	Mol Syst Biol	2011	Yes	No	2	Yes	R_Ec_biomass_iJO1366_core_53p95M	R_Ec_biomass_iJO1366_WT_53p95M	Yes	9.10%
iKF1028	*Burkholderia cenocepacia*	Bacteria	Fang et al.	BMC Syst Biol	2011	No	No	2	No	EX_C9323		No	22.38%
iMB745	*Methanosarcina acetivorans*	Archaea	Benedict et al.	J Bacteriol	2011	No	No	2	No	R_overall		Yes	23.68%
iRC1080	*Chlamydomonas reinhardtii*	Eukaryota	Chang et al.	Mol Syst Biol	2011	No	No	2	No	R_Biomass_Chlamy_hetero	R_Biomass_Chlamy_auto, R_Biomass_Chlamy_mixo	No	26.14%
iRS1563	*Zea mays*	Eukaryota	Saha et al.	PLoS One	2011	No	No	2	No	R_EX_Biomass		No	72.55%
iRS1597	*Arabidopsis thaliana*	Eukaryota	Saha et al.	PLoS One	2011	No	No	2	No	R_EX_Biomass		No	77.36%
iRsp1095	*Rhodobacter sphaeroides*	Bacteria	Saheed Imam et al.	BMC Syst Biol	2011	No	No	2	No	RXN1391	RXN1306	No	13.04%
iVS941_fixed	*Methanosarcina acetivorans*	Archaea	Vinay Satish Kumar et al.	BMC Syst Biol	2011	No	No	2	No	R_Biomass		No	27.31%
iYL1228	*Klebsiella pneumoniae*	Bacteria	Liao et al.	J Bacteriol	2011	No	No	2	Yes	R_Biomass		Yes	21.97%
STM_v1.0	*Salmonella enterica*	Bacteria	Thiele et al.	BMC Syst Biol	2011	Yes	No	2	Yes	R_biomass_iRR1083_metals	R_biomass_iRR1083, R_Ec_biomass_iAF1260_core_59p81M	No	11.00%
VvuMBEL943	*Vibrio vulnificus*	Bacteria	Hyun Uk Kim et al.	Mol Syst Biol	2011	No	No	2	No	R806		No	33.04%
iCce806	*Cyanothece sp. ATCC 51142*	Bacteria	Vu et al.	PLoS Comput Biol	2012	Yes	No	2	Yes	r_CYANOBM		No	27.44%
iJN678	*Synechocystis sp. PCC6803*	Bacteria	Nogales et al.	Proc Natl Acad Sci U S A	2012	No	No	2	No	R_Ec_biomass_SynHetero	R_Ec_biomass_SynAuto, R_Ec_biomass_SynMixo	Yes	15.01%
iLC915	*Pichia pastoris*	Eukaryota	Caspeta et al.	BMC Syst Biol	2012	No	No	2	No	R_r1187		No	27.62%
iSS884	*Pichia stipitis*	Eukaryota	Caspeta et al.	BMC Syst Biol	2012	No	No	2	No	R_biomass		No	32.97%
iSyn731	*Synechocystis sp. PCC6803*	Bacteria	Saha et al.	PLoS One	2012	Yes	No	2	No	Biomass_Auto	Biomass_Hetero, Biomass_Mixo	No	22.95%
RECON 2	*Homo sapiens*	Eukaryota	Thiele et al.	Nature Biotechnology	2012	No	No	2	Yes	R_biomass_reaction		No	30.48%
SpoMBEL1693	*Schizosaccharomyces pombe*	Eukaryota	Sohn et al.	BMC Syst Biol	2012	No	No	1	No	RXNBiomass		No	50.20%
RECON 2.04	*Homo sapiens*	Eukaryota	MATLAB data obtained from Virtual Metabolic Human (vmh.uni.lu)	N/A	2013	No	Yes	N/A	Yes	biomass_reaction		No	30.48%
iCac802	*Clostridium acetobutylicum*	Bacteria	Dash et al.	Biotech for Biofuels	2014	No	No	2	No	R_Biomass		No	55.89%
iMR1_799	*Shewanella oneidensis*	Bacteria	Ong et al.	BMC Systems Biology	2014	Yes	No	2	Yes	R_SO_BIOMASSMACRO_DM_NOATP2		Yes	18.12%
