# Supplementary material for: PSAMM: A Portable System for the Analysis of Metabolic Models
Source: PLoS Comput Biol. 2016 Feb 1;12(2):e1004732. doi: 10.1371/journal.pcbi.1004732 (PMC4734835; doi:10.1371/journal.pcbi.1004732)
Supplement: S4 Table — The model-specific subsystem names in the first column are mapped to the metabolic pathways in the second column. (PDF) [file pcbi.1004732.s006.pdf]

S4 Table. Mapping from model subsystems to metabolic pathways. The model-specific subsystem names in the first column are mapped to the metabolic pathways in the second column.

4 hydroxyphenylacetate catabolism	Amino acid metabolism
Alanine and Aspartate Metabolism	Amino acid metabolism
Alanine Aspartate Metabolism	Amino acid metabolism
alanine biosynthesis	Amino acid metabolism
alanine metabolism	Amino acid metabolism
alanine, aspartate and asparagine biosynthesis	Amino acid metabolism
alanine, aspartate and glutamate metabolism	Amino acid metabolism
alanine/aspartate and asparagine metabolism	Amino acid metabolism
amino acid degradation	Amino acid metabolism
Amino Acid Metabolism	Amino acid metabolism
Amino acid, ala	Amino acid metabolism
aminophosphonate metabolism	Amino acid metabolism
arginine and proline	Amino acid metabolism
Arginine and Proline Metabolism	Amino acid metabolism
arginine biosynthesis	Amino acid metabolism
arginine metabolism	Amino acid metabolism
arginine putriscine and spermidine metabolism	Amino acid metabolism
aromatic amino acid synthesis	Amino acid metabolism
aromatic amino acids	Amino acid metabolism
asparagine degradation	Amino acid metabolism
Asparagine metabolism	Amino acid metabolism
aspartate degradation	Amino acid metabolism
aspartate metabolism	Amino acid metabolism
b-alanine metabolism	Amino acid metabolism
beta-alanine biosynthesis	Amino acid metabolism
beta-Alanine metabolism	Amino acid metabolism
cyanamino acid metabolism	Amino acid metabolism
Cyanophycin Metabolism	Amino acid metabolism
cys biosynthesis	Amino acid metabolism
Cysteine and Methionine Metabolism	Amino acid metabolism
cysteine biosynthesis	Amino acid metabolism
cysteine degradation	Amino acid metabolism
Cysteine Metabolism	Amino acid metabolism
D-alanine metabolism	Amino acid metabolism
d-alanine, d-glutamine and d-glutamate metabolism	Amino acid metabolism
D-arg and D-orn metabolism	Amino acid metabolism
gln biosynthesis	Amino acid metabolism
glutamate and glutamin metabolism	Amino acid metabolism
glutamate and glutamine biosynthesis	Amino acid metabolism
glutamate and glutamine metabolism	Amino acid metabolism
glutamate biosynthesis	Amino acid metabolism
Glutamate Metabolism	Amino acid metabolism
glutamate metabolism (aminosugars metabolism)	Amino acid metabolism
Glutamine Metabolism	Amino acid metabolism

Glutathione Metabolism	Amino acid metabolism
Glycine and Serine Metabolism	Amino acid metabolism
glycine metabolism	Amino acid metabolism
Glycine Serine and Threonine Metabolism	Amino acid metabolism
glycine serine threonine metabolism	Amino acid metabolism
Glycine, Serine and threonine metabolism	Amino acid metabolism
glycine, serine, alanine and threonine metabolism	Amino acid metabolism
Glycine, Serine, and Threonine Metabolism	Amino acid metabolism
histidine biosynthesis	Amino acid metabolism
histidine degradation	Amino acid metabolism
Histidine Metabolism	Amino acid metabolism
homoserine biosynthesis	Amino acid metabolism
ile metabolism	Amino acid metabolism
isoleucine biosynthesis	Amino acid metabolism
leucine degradation	Amino acid metabolism
lysine biosynthesis	Amino acid metabolism
lysine degradation	Amino acid metabolism
Lysine Metabolism	Amino acid metabolism
lysine/ threonine biosynthesis	Amino acid metabolism
methionine and cysteine metabolism	Amino acid metabolism
methionine biosynthesis	Amino acid metabolism
Methionine Metabolism	Amino acid metabolism
methionine salvage pathway	Amino acid metabolism
Other Amino Acid Metabolism	Amino acid metabolism
phenylalanine biosynthesis	Amino acid metabolism
Phenylalanine Metabolism	Amino acid metabolism
phenylalanine tyrosine and tryptophan biosynthesis	Amino acid metabolism
phenylalanine tyrosine tryptophan biosynthesis	Amino acid metabolism
Phenylalanine Tyrosine Tryptophan Metabolism	Amino acid metabolism
phenylalanine, tyrosine and tryptophan biosynthesis	Amino acid metabolism
phenylalanine, tyrosine and tryptophan biosynthesis (aromatic amino acids)	Amino acid metabolism
phenylalanine, tyrosine, and tryptophan biosynthesis (aromatic amino acids)	Amino acid metabolism
phenylalanine, tyrosine, tryptophan biosynthesis	Amino acid metabolism
phenylalanine/phenylacetate degradation	Amino acid metabolism
polyamine synthesis	Amino acid metabolism
Proline Biosynthesis	Amino acid metabolism
proline degradation	Amino acid metabolism
proline synthesis	Amino acid metabolism
Selenoamino acid metabolism	Amino acid metabolism
ser, gly biosynthesis	Amino acid metabolism
serine metabolism	Amino acid metabolism
serine, glycine and threonine biosynthesis	Amino acid metabolism
shikimate cycle	Amino acid metabolism
sulfur cysteine and methionine metabolism	Amino acid metabolism
Taurine and hypotaurine metabolism	Amino acid metabolism
Taurine Metabolism	Amino acid metabolism
Thiamine Metabolism	Amino acid metabolism
Threonine and Lysine Metabolism	Amino acid metabolism

threonine and serine degradation	Amino acid metabolism
threonine metabolism	Amino acid metabolism
tryptophan biosynthesis	Amino acid metabolism
tryptophan degradation	Amino acid metabolism
Tryptophan metabolism	Amino acid metabolism
Tyr, Phe, Trp Biosynthesis	Amino acid metabolism
tyrosine biosynthesis	Amino acid metabolism
tyrosine degradation	Amino acid metabolism
Tyrosine metabolism	Amino acid metabolism
Tyrosine Tryptophan and Phenylalanine Metabolism	Amino acid metabolism
Tyrosine, Tryptophan, and Phenylalanine Metabolism	Amino acid metabolism
Urea Cycle	Amino acid metabolism
Urea Cycle and Amino Acid Metabolisms	Amino acid metabolism
urea cycle and metabolism of amino groups	Amino acid metabolism
Urea cycle/amino group metabolism	Amino acid metabolism
val metabolism	Amino acid metabolism
valine and leucine biosynthesis	Amino acid metabolism
valine leucine and isoleucine biosynthesis	Amino acid metabolism
Valine Leucine and Isoleucine Metabolism	Amino acid metabolism
valine leucine isoleucine metabolism	Amino acid metabolism
valine, leucine and isoleucine biosynthesis	Amino acid metabolism
valine, leucine and isoleucine degradation	Amino acid metabolism
valine, leucine, and isoleucine biosynthesis	Amino acid metabolism
valine, leucine, and isoleucine degradation	Amino acid metabolism
Valine, Leucine, and Isoleucine Metabolism	Amino acid metabolism
1 2 Propanediol Catabolism	Carbon metabolism
3-hydroxybenzoate degradation	Carbon metabolism
acetate/acetaldehyde/ethanol metabolism	Carbon metabolism
Alternate Carbon Metabolism	Carbon metabolism
Alternate Carbon source	Carbon metabolism
Amino Sugar and Nucleotide Sugar Metabolism	Carbon metabolism
Aminosugar Metabolism	Carbon metabolism
aminosugars metabolism	Carbon metabolism
Anaplerotic Reactions	Carbon metabolism
Anaplerotic reactions	Carbon metabolism
Arabinose Metabolism	Carbon metabolism
Aromatic Acid Breakdown	Carbon metabolism
Ascorbate and Aldarate Metabolism	Carbon metabolism
Benzoate degradation	Carbon metabolism
Butanoate Metabolism	Carbon metabolism
C5 Branched dibasic acid metabolism	Carbon metabolism
C5-Branched dibasic acid metabolism	Carbon metabolism
c5-branched dibasic acid metabolsim	Carbon metabolism
Carbohydrate Metabolism	Carbon metabolism
Carbon Fixation	Carbon metabolism
Central Metabolism	Carbon metabolism
Citrate Cycle (TCA)	Carbon metabolism
Citrate Cycle TCA	Carbon metabolism
Citric Acid Cycle	Carbon metabolism
Complex Alcohol Metabolism	Carbon metabolism

flourene degradation	Carbon metabolism
Fructose and mannose metabolism	Carbon metabolism
Galactose metabolism	Carbon metabolism
Glucosamine Metabolism	Carbon metabolism
Glycolysis	Carbon metabolism
Glycolysis/Gluconeogenesis	Carbon metabolism
GlycolysisGluconeogenesis	Carbon metabolism
Glyoxylate and Dicarboxylate Metabolism	Carbon metabolism
Glyoxylate Metabolism	Carbon metabolism
Inositol Metabolism	Carbon metabolism
Inositol Phosphate Metabolism	Carbon metabolism
Mannitol Metabolism	Carbon metabolism
methylcitrate cycle	Carbon metabolism
Methylglyoxal Metabolism	Carbon metabolism
Nucleotide Sugar Metabolism	Carbon metabolism
one carbon metabolism	Carbon metabolism
one carbon pool by folate	Carbon metabolism
pentose and glucoronate interconverstion	Carbon metabolism
Pentose and Glucuronate Interconversions	Carbon metabolism
Pentose Phosphate Pathway	Carbon metabolism
Photosynthesis	Carbon metabolism
Propanoate Metabolism	Carbon metabolism
Pyruvate Metabolism	Carbon metabolism
Pyruvate metabolism	Carbon metabolism
Starch and Sucrose Metabolism	Carbon metabolism
Sugar Metabolism	Carbon metabolism
Xylose Metabolism	Carbon metabolism
biotin biosynthesis	Cofactors and vitamins metabolism
Biotin Metabolism	Cofactors and vitamins metabolism
CoA Biosynthesis	Cofactors and vitamins metabolism
CoA Catabolism	Cofactors and vitamins metabolism
coa synthesis	Cofactors and vitamins metabolism
Coenzyme A Biosynthesis	Cofactors and vitamins metabolism
Coenzyme B Biosynthesis	Cofactors and vitamins metabolism
Coenzyme M Biosynthesis	Cofactors and vitamins metabolism
Cofactor and Prostehtic Group Biosynthesis	Cofactors and vitamins metabolism
Cofactor and Prosthetic Group Biosynthesis	Cofactors and vitamins metabolism
Cofactor Metabolism	Cofactors and vitamins metabolism
Cofactors and vitamins metabolism Biosynthesis	Cofactors and vitamins metabolism
CYP Metabolism	Cofactors and vitamins metabolism
folate biosynthesis	Cofactors and vitamins metabolism
Folate Metabolism	Cofactors and vitamins metabolism
folate one-carbon pool	Cofactors and vitamins metabolism
folate synthesis	Cofactors and vitamins metabolism
Heme Biosynthesis	Cofactors and vitamins metabolism
Heme Degradation	Cofactors and vitamins metabolism
heme synthesis	Cofactors and vitamins metabolism
nad and nadp biosynthesis	Cofactors and vitamins metabolism
nad and nadp conversion	Cofactors and vitamins metabolism
NAD Biosynthesis	Cofactors and vitamins metabolism

NAD Metabolism	Cofactors and vitamins metabolism
nadp and nad degradation	Cofactors and vitamins metabolism
nicotinate and nicotinamide	Cofactors and vitamins metabolism
nicotinate and nicotinamide biosynthesis	Cofactors and vitamins metabolism
Nicotinate and Nicotinamide Metabolism	Cofactors and vitamins metabolism
nicotinate biosynthesis	Cofactors and vitamins metabolism
nicotinic acid pathway	Cofactors and vitamins metabolism
nicotinic acid synthesis from trp	Cofactors and vitamins metabolism
Pantothenate and CoA Biosynthesis	Cofactors and vitamins metabolism
Pantothenate and CoA Metabolism	Cofactors and vitamins metabolism
Phylloquinone Biosynthesis	Cofactors and vitamins metabolism
Plastoquinone Biosynthesis	Cofactors and vitamins metabolism
Polyprenyl Metabolism	Cofactors and vitamins metabolism
Porphyrin and Chlorophyll Metabolism	Cofactors and vitamins metabolism
Porphyrin Metabolism	Cofactors and vitamins metabolism
Pyridoxine Metabolism	Cofactors and vitamins metabolism
Quinone Biosynthesis	Cofactors and vitamins metabolism
Riboflavin Metabolism	Cofactors and vitamins metabolism
Tetrahydramethanopterin Biosynthesis	Cofactors and vitamins metabolism
Tetrahydrobiopterin	Cofactors and vitamins metabolism
tetrahydrobiopterin metabolism	Cofactors and vitamins metabolism
thf metabolism	Cofactors and vitamins metabolism
thiamin biosynthesis	Cofactors and vitamins metabolism
thiamine (vitamin b1) metabolism	Cofactors and vitamins metabolism
thiamine biosynthesis	Cofactors and vitamins metabolism
Ubiquinone Biosynthesis	Cofactors and vitamins metabolism
Ubiquinone Metabolism	Cofactors and vitamins metabolism
ubiquinone synthesis	Cofactors and vitamins metabolism
Vitamin A Metabolism	Cofactors and vitamins metabolism
Vitamin B12 Metabolism	Cofactors and vitamins metabolism
vitamin b2 metabolism	Cofactors and vitamins metabolism
Vitamin B6 Metabolism	Cofactors and vitamins metabolism
vitamin c metabolism	Cofactors and vitamins metabolism
Vitamin D	Cofactors and vitamins metabolism
vitamin d metabolism	Cofactors and vitamins metabolism
vitamin e metabolism	Cofactors and vitamins metabolism
Vitamins & Cofactor Biosynthesis	Cofactors and vitamins metabolism
cytochrome metabolism	Energy metabolism
Energy Metabolism	Energy metabolism
Hydrogen Metabolism	Energy metabolism
Methane Metabolism	Energy metabolism
Methanogenesis	Energy metabolism
Nitrogen Metabolism	Energy metabolism
Oxidative Phosphorylation	Energy metabolism
Redox Metabolism	Energy metabolism
ros detoxification	Energy metabolism
Sulfur Metabolism	Energy metabolism
Blood Group Biosynthesis	Glycan metabolism
blood group synthesis	Glycan metabolism
Chondroitin / heparan sulfate biosynthesis	Glycan metabolism

Chondroitin sulfate degradation	Glycan metabolism
chondroitin synthesis	Glycan metabolism
glycan biosynthesis	Glycan metabolism
Glycoprotein Metabolism	Glycan metabolism
Glycosylphosphatidylinositol (GPI)-anchor biosynthesis	Glycan metabolism
Heparan sulfate degradation	Glycan metabolism
high-mannose type n-glycan biosynthesis	Glycan metabolism
Hyaluronan Metabolism	Glycan metabolism
Keratan sulfate biosynthesis	Glycan metabolism
Keratan sulfate degradation	Glycan metabolism
keratan sulfate synthesis	Glycan metabolism
Lipopolysaccharide Biosynthesis	Glycan metabolism
Lipopolysaccharide Biosynthesis / Recycling	Glycan metabolism
Lipopolysaccharide Biosynthesis Recycling	Glycan metabolism
Murein Biosynthesis	Glycan metabolism
Murein lpp biosynthesis	Glycan metabolism
Murein Recycling	Glycan metabolism
N-Glycan Biosynthesis	Glycan metabolism
N-Glycan Degradation	Glycan metabolism
n-glycan metabolism	Glycan metabolism
n-glycan synthesis	Glycan metabolism
O-Glycan Biosynthesis	Glycan metabolism
o-glycan synthesis	Glycan metabolism
O6 antigen synthesis	Glycan metabolism
other glycan degradation	Glycan metabolism
Peptidoglycan Biosynthesis	Glycan metabolism
Peptidoglycan Metabolism	Glycan metabolism
phosphatidylinositol phosphate metabolism	Glycan metabolism
alpha-linolenic acid metabolism	Lipid metabolism
arachidonic acid metabolism	Lipid metabolism
Bile Acid Biosynthesis	Lipid metabolism
bile acid synthesis	Lipid metabolism
biosynthesis of unsaturated fatty acids	Lipid metabolism
biosynthesis of very long fatty acids	Lipid metabolism
Carnitine shuttle	Lipid metabolism
Cell Envelope Biosynthesis	Lipid metabolism
Cholesterol Metabolism	Lipid metabolism
Fatty Acid Biosynthesis	Lipid metabolism
Fatty acid activation	Lipid metabolism
Fatty Acid Biosynthesis	Lipid metabolism
Fatty Acid Degradation	Lipid metabolism
Fatty acid elongation	Lipid metabolism
Fatty Acid Metabolism	Lipid metabolism
Fatty acid oxidation	Lipid metabolism
Fatty acid oxidation, peroxisome	Lipid metabolism
Fatty Acid Synthesis	Lipid metabolism
Glycerolipid Metabolism	Lipid metabolism
Glycerophospholipid Metabolism	Lipid metabolism
Glycosphingolipid metabolism	Lipid metabolism
linoleate metabolism	Lipid metabolism

Lipid & Cell Wall Metabolism	Lipid metabolism
lipid and cell wall metabolism	Lipid metabolism
lipid biosynthesis	Lipid metabolism
lipid biosynthesis proteins	Lipid metabolism
Lipid Metabolism	Lipid metabolism
lipoate metabolism	Lipid metabolism
Membrane Lipid Metabolism	Lipid metabolism
Membrane Metabolism	Lipid metabolism
Phospholipid Biosynthesis	Lipid metabolism
phospholipid degradation	Lipid metabolism
Phospholipid Metabolism	Lipid metabolism
Sphingolipid Metabolism	Lipid metabolism
squalene and cholesterol synthesis	Lipid metabolism
Steroid Metabolism	Lipid metabolism
Sterol Biosynthesis	Lipid metabolism
Sterol Metabolism	Lipid metabolism
synthesis of unsaturated fatty acids	Lipid metabolism
Triacylglycerol Synthesis	Lipid metabolism
atp de novo synthesis	Nucleic acid metabolism
IMP Biosynthesis	Nucleic acid metabolism
Nucleic acid degradation	Nucleic acid metabolism
nucleotide interconversion	Nucleic acid metabolism
Nucleotide Metabolism	Nucleic acid metabolism
Nucleotide Salvage Pathway	Nucleic acid metabolism
Nucleotide Salvage Pathways	Nucleic acid metabolism
Nucleotides	Nucleic acid metabolism
Purine and Pyrimidine Biosynthesis	Nucleic acid metabolism
Purine and Pyrimidine Metabolism	Nucleic acid metabolism
Purine Catabolism	Nucleic acid metabolism
Purine Metabolism	Nucleic acid metabolism
Purine Metabolism, Transport	Nucleic acid metabolism
purine synthesis	Nucleic acid metabolism
Pyrimidine Biosynthesis	Nucleic acid metabolism
Pyrimidine Catabolism	Nucleic acid metabolism
Pyrimidine Metabolism	Nucleic acid metabolism
pyrimidine synthesis	Nucleic acid metabolism
tRNA Charging	Nucleic acid metabolism
alkaloid biosynthesis i	Secondary and Xenobiotics metabolism
Alkaloid biosynthesis II	Secondary and Xenobiotics metabolism
alkaloid synthesis	Secondary and Xenobiotics metabolism
androgen and estrogen synthesis and metabolism	Secondary and Xenobiotics metabolism
biosynthesis of siderophore group nonribosomal peptides	Secondary and Xenobiotics metabolism
biosynthesis of siderophore group nonribosomal petides	Secondary and Xenobiotics metabolism
Carotenoid Biosynthesis	Secondary and Xenobiotics metabolism
Eicosanoid Metabolism	Secondary and Xenobiotics metabolism
Limonene and pinene degradation	Secondary and Xenobiotics metabolism
penicillin breakdown	Secondary and Xenobiotics metabolism
penincillin biosynthesis	Secondary and Xenobiotics metabolism
Stilbene, coumarine and lignin biosynthesis	Secondary and Xenobiotics metabolism
stilbene, coumarine and lignin synthesis	Secondary and Xenobiotics metabolism

Terpenoid Backbone Biosynthesis	Secondary and Xenobiotics metabolism
tropane, piperidine and pyridine alkaloid biosynthesis	Secondary and Xenobiotics metabolism
xenobiotics metabolism	Secondary and Xenobiotics metabolism
carrier-mediated transport across the cytoplasmatic membrane	Transport and exchange
carrier-mediated transport across the mitochondrial membrane	Transport and exchange
er transport	Transport and exchange
Exchange	Transport and exchange
exchange reactions	Transport and exchange
exchange/demand reaction	Transport and exchange
golgi transport	Transport and exchange
golgi trasnport	Transport and exchange
Inorganic Ion Transport and Metabolism	Transport and exchange
mitochondrial transport	Transport and exchange
non-carrier-mediated transport across the cytoplasmastic membrane	Transport and exchange
non-carrier-mediated transport across the mitochondrial membrane	Transport and exchange
peroxisomal transport	Transport and exchange
peroxisomal transport	Transport and exchange
peroxisomal trasnport	Transport and exchange
putative transporters	Transport and exchange
Tranport	Transport and exchange
Tranport, Inner Membrane	Transport and exchange
Tranpsort, Inner Membrane	Transport and exchange
Transport	Transport and exchange
Transport Endoplasmic Reticular	Transport and exchange
Transport Extracellular	Transport and exchange
Transport Golgi Apparatus	Transport and exchange
Transport Inner Membrane	Transport and exchange
Transport Mitochondrial	Transport and exchange
Transport Nuclear	Transport and exchange
transport of fab	Transport and exchange
Transport Outer Membrane	Transport and exchange
Transport Outer Membrane Porin	Transport and exchange
Transport Peroxisomal	Transport and exchange
transport plasma membrane	Transport and exchange
transport reaction	Transport and exchange
Transport Vacuolar	Transport and exchange
transport, abc system	Transport and exchange
Transport, Endoplasmic Reticular	Transport and exchange
Transport, Extracellular	Transport and exchange
Transport, Golgi Apparatus	Transport and exchange
Transport, Inner Membrane	Transport and exchange
Transport, Lysosomal	Transport and exchange
Transport, Mitochondrial	Transport and exchange
transport, mitochondrial membrane	Transport and exchange
Transport, Nuclear	Transport and exchange
Transport, Outer Membrane	Transport and exchange
Transport, Outer Membrane Porin	Transport and exchange
Transport, Peroxisomal	Transport and exchange
transport, peroxisomal membrane	Transport and exchange
transport, plasma membrane	Transport and exchange

transport, plasma membrane (alcohols and acids)	Transport and exchange
transport, plasma membrane (amino acids)	Transport and exchange
transport, plasma membrane (carbohydrates)	Transport and exchange
transport, plasma membrane (nucleotides)	Transport and exchange
transport, plasma membrane (other compounds)	Transport and exchange
Transport, Vacuolar	Transport and exchange
vacuolar transport	Transport and exchange
vacuolar trasnport	Transport and exchange
--	Unassigned
.	Unassigned
Biomass	Unassigned
biomass composition	Unassigned
biomass formation	Unassigned
biomass production	Unassigned
biomass reactions - biomass formation	Unassigned
biomass reactions - cell wall composition	Unassigned
biomass reactions - dna formation	Unassigned
biomass reactions - lipids	Unassigned
biomass reactions - protein formation	Unassigned
biomass reactions - rna formation	Unassigned
biomass reactions - small molecules pool	Unassigned
biomassobjective	Unassigned
blankfield	Unassigned
demand	Unassigned
dietary fiber binding	Unassigned
general reaction	Unassigned
Growth	Unassigned
growthrate	Unassigned
Macromolecule Synthesis	Unassigned
maintenance	Unassigned
maintenance requirements (non-growth associated)	Unassigned
metabolic pathways	Unassigned
Miscellaneous	Unassigned
n - others	Unassigned
None	Unassigned
Other	Unassigned
Others	Unassigned
putative	Unassigned
R Group Synthesis	Unassigned
Salvage Pathway	Unassigned
spontaneous	Unassigned
Translation	Unassigned
Unassigned	Unassigned
Unknown	Unassigned
