# Supplementary material for: PSAMM: A Portable System for the Analysis of Metabolic Models
Source: PLoS Comput Biol. 2016 Feb 1;12(2):e1004732. doi: 10.1371/journal.pcbi.1004732 (PMC4734835; doi:10.1371/journal.pcbi.1004732)
Supplement: S1 Text — (PDF) [file pcbi.1004732.s007.pdf]

S1 Text: Description of the PSAMM YAML model format.
The YAML model format allows the use of the human readable YAML language to represent the many aspects of genome scale metabolic models in separate, easily interpretable components. The YAML model format that is used with PSAMM allows the users to easily modify various aspects of the model during model curation and simulation and also provides a way for the users to separate the definition of simulation problems from the representation of static model components such as the list of metabolites and reactions.

The YAML model format consists of one central module and five annotation modules, among the annotation modules three are required and two are optional. The list of modules in the YAML model representation are given below:

- **model.yaml**: central model definition
- **reactions**: list of reactions and annotated features
- **compounds** (optional): list of metabolites and annotated features
- **media**: boundary conditions
- **model** (optional): subset of reaction IDs that are included in the current model
- **limits** (optional): list of flux bounds that deviate from the default values of [-1000,1000] for reversible reactions or [0,1000] for irreversible reactions

The annotation modules are stored in individual files and referenced in the central module using an *include* function in YAML. Alternatively, content of the annotation modules can be incorporated directly into the central model file to form a unified model definition. However, this latter approach is not recommended because it diminishes the modularity of the YAML format.

1. Central model definitions in *model.yaml*
The *model.yaml* file serves as a hub that links the various annotation modules into the definition of a YAML model. This central file allows the users to easily change what files are included in any particular simulation by simply altering the file names referenced in the *include* function. The model.yaml file is set up in the following way:

```
# ----- Define the name of the model (optional)
name: <Model Name>

# ----- Define the biomass reaction ID (optional)
# ----- The biomass reaction is used as a default objective function in FBA simulations
biomass: <Biomass Reaction ID>
```

```
# ----- Define the default flux value (optional).
# ----- If the default flux is not defined, use 1000 as the default
default_flux_limits: <Value>

# ----- Define the list of reactions and reaction features
# ----- The reactions can be presented in either a YAML format or a table format
reactions:
- include: reaction_list_1.yaml
- include: reaction_list_2.tsv

# ----- Define the list of metabolites and metabolite features (optional)
# ----- The metabolites can be presented in either a YAML format or a table format
compounds:
- include: metabolite_list_1.yaml
- include: metabolite_list_2.tsv

# ----- Define the boundary conditions
# ----- Represented as a list of boundary compounds and their exchange reactions
media:
- include: medium_1.yaml

# ----- Define a subset of reaction IDs to be included in the current simulation (optional)
model:
- include: reaction_subset.txt

# ----- Define the limits of flux bounds (optional)
# ----- The limits are specified only when flux bounds deviate from the default value
limits:
- include: limits.yaml
```

Note: The following rule will apply for all the files in the YAML model: (1) The lines start with # are comment lines, which can be kept as it is or removed from the central model file. PSAMM will automatedly ignore comments when parsing YAML models. (2) The content marked with <> in this description should be replaced with the real content of

the model. For example, if a model name is “E.coli Textbook Model”, it will be defined in model.yaml with the following line:

name: E.coli Textbook Model

2. **Define the static model content in reactions and compounds modules**
    a. **Reactions**

The reaction module can be formatted either as a YAML reaction file or as a annotation table that includes all the reaction features. It contains all the relevant information on the metabolic reactions in the model and is designed to be both human readable and machine-readable. The YAML format of the reactions module is formatted in the following way:

- **id**: Reaction_ID
equation: ‘|cpd_1[c]| => |cpd_2[c]|’
name: <Reaction Name>
subsystem: <Subsystem Identifier>
genes: <Gene Information>
ec: <EC Number>

The only required components for each metabolic reaction are the id and equation (highlighted in bold above). The other attributes can be included or left out as desired by the user. Non-standard reaction attributes can also be included for each reaction by adding in a new line with the user-defined attribute name followed by the data. For example if a user wanted to add in a designation that showed if the reaction was essential to the model or not, then they could add in an essential attribute as long as it was kept in the indented information under the reaction ID. This allows flexibility with the annotation of the reactions as well as providing an easier way to connect all of the relevant data.

The flexibility of the YAML format can also allow the user to specify reactions in different ways. Some metabolic reactions can involve many metabolites and can be cumbersome to read in one line. The YAML model format allows the user to specify these metabolites and their stoichiometric values in individual lines. For example a reaction may appear as follows:

- **id**: Reaction_ID
equation:
reversible: no
left:
- id: cpd_1[c]
value: 1
right:
- id: cpd_2[c]

value: 2

This format of representing reaction equations allows the user to designate reversibility of the reaction and then the compounds on the left and right side of the metabolic reaction. This flexibility allows users to easily track the content of long reaction equations.

Gene associations can be included for reactions in the genes property. Gene association rules can be formatted as logical strings for reactions with multiple genes. This can include and statements for reactions that may require multiple gene products and or statements for reactions that may have multiple different genes that code for proteins of the same function. This information can be used for gene essentiality analyses. The following are examples of how this kind of information can be added to a reaction.

- **id**: Reaction_ID
  equation: ‘|cpd_1[c]| => |cpd_2[c]|’
  genes: gene_0001 and gene_0002

- id: Reaction_ID
  equation: ‘|cpd_3[c]| => |cpd_4[c]|’
  genes: gene_0003 and (gene_0004 or gene_0005)

The reaction list can also be represented in a tab-delimited table format (TSV format), where each row contains the reaction ID, reaction equation, and other data columns. A header must be included to specify the name of each column. The column equation will be parsed based on conventions set up in the ModelSEED database.

b. Compounds

The compounds module is defined by metabolite IDs followed by their various attributes. The YAML format of the compounds module is formatted in the following way:

- **id**: cpd_1
  name: <Metabolite Name>
  formula: <Metabolite Formula>
  charge: <Metabolite Charge>
  kegg: <Metabolite KEGG ID>
  cas: <Metabolite CAS number>

The compounds module is optional in a YAML formatted model, but its inclusion allows users to have more human friendly output from constraint-based analysis as well as allowing the use of the formula consistency and charge consistency checking functions that are part of PSAMM. This format also allows for the inclusion of user-defined

attributes by adding in other attribute items under the compound ID. The additional attributes should be included with a definition of the property name followed by the data. This allows for more flexibility and the inclusion of all relevant information in one place.

The compound list can also be represented in a tab-delimited table format (TSV format), where the rows contain the metabolite ID and other data columns. Similar to the reaction tables, a header must be included in the compound table to specify the name of each column.

3. Define the dynamic simulation settings in other modules

The reactions and compounds modules are the central components of the static model definition. In the YAML format these modules are separated from the definition of simulation problems, which are specified in combinations of the media, model, and limits modules. This allows for the modification of the simulation problems while core components of the model remains constant. While the users are required to define a media module in any simulation problems, the model and the limits modules are optional.

a. Media:

The media module is where the boundary conditions for the simulation are defined. This module is formatted with the IDs of the exchange compounds and the fluxes of the exchange reactions, with an optional compartment definition that sets the cellular compartment of the exchange compounds. By default, this is the extracellular space. A media file can be formatted in the following way.

compartment: <Compartment_Designation (e.g. C_e)>
compounds:
- **id**: cpd_1
  lower: <Value>
  upper: <Value>

This only required information in the media module is the compound ID. if only this is provided the boundary condition will have a maximum and minimum flux set to the default flux limits of the model. The user can include lower and upper bounds which allows for the designation of sinks and sources. This media component is also flexible and allows for the inclusion of user-defined attributes by the designation of an attribute name followed by the information.

The media module can be also represented in a tab-delimited table format, which should start with four columns, which indicates the compound ID, compartment, lower bounds, and upper bounds. The table should include a header row that defines the name of each column. An example is shown below:

id	compartment	lower	upper
akg	e	0	400
glc-D	e	-10	-

Additional features can be included in the media table to indicate classifications of the exchange compounds (e.g. carbon sources, electron acceptors, etc.) A dash sign (-) can be used in the columns for the lower and upper bounds to indicate default values. The sign can also be left out if it is at the end of a row.

b. Model

The model definition module is an optional module that contains a list of reaction IDs. If this module is included in the central model file, only the reactions specified in the model list will be used in constraint-based simulations. If not, all reactions listed in the reaction module will be included in the simulation. The model file is formatted as a list of reaction IDs with each new ID being on a new line. A sample of a model definition would be as follows:

```
ID_1
ID_2
ID_3
…
```

c. Limits:

The limits module is where the flux limits for individual reactions can be set for the simulation. The limits component consists of a reaction ID followed by optional upper and lower limits. If the reactions will have the default flux limits for the model then it is not necessary to designate the limits for the reaction. A typical limits component will be formatted in the following way:

- **reaction**: RXN_ID
  upper: Value
  lower: Value

The limits module is also flexible and allows for the inclusion of user defined attributes. Similar to the other modules, The limits module can also be represented in a tab-delimited table format, which includes three predefined columns (i.e. Reaction ID, lower , upper) and can be extended to include other annotation information. An example of the limits module in the table format is shown below:

```
reaction ID    lower  upper
# ----- Make ADE2t irreversible by imposing a lower bound of 0
ADE2t          0      -
# ----- Only allow limited flux on ADK1
ADK1           -10    10
```

Overall the YAML model format allows for a modular definition of the model content. It supports the central organization of separated model components while still maintaining the independence between static model definitions and the dynamic simulation settings. The format is flexible for integrating heterogeneous annotation data and is highly customizable with respect to the organization of user-defined annotations.
