# Supplementary material for: PSAMM: A Portable System for the Analysis of Metabolic Models
Source: PLoS Comput Biol. 2016 Feb 1;12(2):e1004732. doi: 10.1371/journal.pcbi.1004732 (PMC4734835; doi:10.1371/journal.pcbi.1004732)
Supplement: S2 Text — The original version cannot provide a non-zero flux in FBA simulations because the periplasmic cardiolipin compounds (M_clpn120_p, M_clpn160_p, M_clpn161_p, M_clpn180_p, M_clpn181_p) were mislabeled as cytosolic compounds in the SBML model. The updated model correctly incorporates the mislabeled compounds and is able to produce a non-zero flux on biomass. The lines are color coded to highlight manual corrections made to the model. Highlighted in red are the lines removed, and highlighted in green are the lines added from the original version to the new version. (PDF) [file pcbi.1004732.s008.pdf]

S2 Text: Comparison of the original version [58] and the fixed version of iJN746 using the *git diff* function in the Git version control system. The original version cannot provide a non-zero flux in FBA simulations because the periplasmic cardiolipin compounds (M_clpn120_p, M_clpn160_p, M_clpn161_p, M_clpn180_p, M_clpn181_p) were mislabeled as cytosolic compounds in the SBML model. The updated model correctly incorporates the mislabeled compounds and is able to produce a non-zero flux on biomass. The lines are color coded to highlight manual corrections made to the model. Highlighted in red are the lines removed, and highlighted in green are the lines added from the original version to the new version.

```
diff --git a/sbml/iJN746/reactions.yaml b/sbml/iJN746/reactions.yaml
index d69f70b..f8a75fe 100644
--- a/sbml/iJN746/reactions.yaml
+++ b/sbml/iJN746/reactions.yaml
@@ -851,8 +851,9 @@
      + (0.035) |M_ptrc_c[C_c]| + (0.205) |M_ser_DASH_L_c[C_c]| + (0.0005) |M_sheme_c[C_c]|
      + (0.000003) |M_succoa_c[C_c]| + (0.241) |M_thr_DASH_L_c[C_c]| + (0.054) |M_trp_DASH_L_c[C_c]|
      + (0.131) |M_tyr_DASH_L_c[C_c]| + (0.003) |M_udpg_c[C_c]| + (0.136) |M_utp_c[C_c]|
-    + (0.402) |M_val_DASH_L_c[C_c]| => (45.5608) |M_adp_c[C_c]| + (45.56035) |M_h_c[C_c]|
-    + (45.5628) |M_pi_c[C_c]| + (0.7302) |M_ppi_c[C_c]|
+    + (0.402) |M_val_DASH_L_c[C_c]| + (0.0005) |M_clpn120_p[C_p]| + (0.0005) |M_clpn160_p[C_p]|
+    + (0.0005) |M_clpn161_p[C_p]| + (0.0005) |M_clpn180_p[C_p]| + (0.0005) |M_clpn181_p[C_p]|
+     => (45.5608) |M_adp_c[C_c]| + (45.56035) |M_h_c[C_c]| + (45.5628) |M_pi_c[C_c]| + (0.7302) |M_ppi_c[C_c]|
 - id: R_CACOAHA
   name: Enoyl-CoA hydratase/aldolase
   equation: '|M_caffcoa_c[C_c]| + |M_h2o_c[C_c]| => |M_34dhbald_c[C_c]| + |M_accoa_c[C_c]|'
```
