# Supplementary material for: PSAMM: A Portable System for the Analysis of Metabolic Models
Source: PLoS Comput Biol. 2016 Feb 1;12(2):e1004732. doi: 10.1371/journal.pcbi.1004732 (PMC4734835; doi:10.1371/journal.pcbi.1004732)
Supplement: S3 Text — The original version cannot provide a non-zero flux in FBA simulations, while the fixed version can. This was determined to be due to all of the exchange reactions being set to have a lower bound of zero thus preventing uptake of any nutrients. The exchange reactions were altered to reflect the boundary conditions stated in the supplemental materials of the original paper [66]. The lines are color coded to highlight the changes between the two versions. Highlighted in red are the lines removed, and highlighted in green are the lines added from the original version to the new version. (PDF) [file pcbi.1004732.s009.pdf]

S3 Text: Comparison of the original version and the fixed version of the model iRsp1095 [66] model using the *git diff* function in the Git version control system. The original version cannot provide a non-zero flux in FBA simulations, while the fixed version can. This was determined to be due to the all of the exchange reactions being set to have a lower bound of zero thus preventing uptake of any nutrients. The exchange reactions were altered to reflect the boundary conditions stated in the supplemental materials of the original paper [66]. The lines are color coded to highlight the changes between the two versions. Highlighted in red are the lines removed, and highlighted in green are the lines added from the original version to the new version.

```
diff --git a/sbml/iRsp1095/medium.yaml b/sbml/iRsp1095/medium.yaml
index a6acaca..7c6d0d7 100644
--- a/sbml/iRsp1095/medium.yaml
+++ b/sbml/iRsp1095/medium.yaml
@@ -51,7 +51,7 @@ compounds:
   lower: 0
 - id: CPD0829
   reaction: RXN0192
-  lower: 0
+  lower: -1000
 - id: CPD0835
   reaction: RXN0193
   lower: 0
@@ -63,7 +63,7 @@ compounds:
   lower: 0
 - id: CPD0730
   reaction: RXN0196
-  lower: 0
+  lower: -1000
 - id: CPD0732
   reaction: RXN0197
   lower: 0
@@ -111,7 +111,7 @@ compounds:
   lower: 0
 - id: CPD0771
   reaction: RXN0213
-  lower: 0
+  lower: -1000
 - id: CPD0773
   reaction: RXN0214
   lower: 0
@@ -126,7 +126,7 @@ compounds:
   lower: 0
 - id: CPD0875
   reaction: RXN0219
-  lower: 0
+  lower: -1000
 - id: CPD0878
   reaction: RXN0220
   lower: 0
@@ -138,7 +138,7 @@ compounds:
   lower: 0
 - id: CPD0885
   reaction: RXN0223
-  lower: 0
+  lower: -1000
   lower: 0
 - id: CPD0829
   reaction: RXN0192
-  lower: 0
+  lower: -1000
 - id: CPD0835
```

```diff
   reaction: RXN0193
   lower: 0
@@ -63,7 +63,7 @@ compounds:
   lower: 0
 - id: CPD0730
   reaction: RXN0196
-  lower: 0
+  lower: -1000
 - id: CPD0732
   reaction: RXN0197
   lower: 0
@@ -111,7 +111,7 @@ compounds:
   lower: 0
 - id: CPD0771
   reaction: RXN0213
-  lower: 0
+  lower: -1000
 - id: CPD0773
   reaction: RXN0214
   lower: 0
@@ -126,7 +126,7 @@ compounds:
   lower: 0
 - id: CPD0875
   reaction: RXN0219
-  lower: 0
+  lower: -1000
 - id: CPD0878
   reaction: RXN0220
   lower: 0
@@ -138,7 +138,7 @@ compounds:
   lower: 0
 - id: CPD0885
   reaction: RXN0223
-  lower: 0
+  lower: -1000
 - id: CPD0887
   reaction: RXN0224
   lower: 0
@@ -189,7 +189,7 @@ compounds:
   lower: 0
 - id: CPD1074
   reaction: RXN1101
-  lower: 0
+  lower: -1000
 - id: CPD1076
   reaction: RXN1104
   lower: 0
@@ -243,7 +243,7 @@ compounds:
   lower: 0
 - id: CPD1114
   reaction: RXN1158
-  lower: 0
+  lower: -1000
 - id: CPD1116
   reaction: RXN1161
   lower: 0
@@ -378,10 +378,10 @@ compounds:
   lower: 0
 - id: CPD1211
   reaction: RXN1326
-  lower: 0
```

```diff
+   lower: -1000
 - id: CPD1213
   reaction: RXN1329
-   lower: 0
+   lower: -1000
 - id: CPD1214
   reaction: RXN1331
   lower: 0
@@ -393,7 +393,7 @@ compounds:
   lower: 0
 - id: CPD0734
   reaction: RXN1354
-   lower: 0
+   lower: -1000
 - id: CPD1231
   reaction: RXN1359
   lower: 0
```
