# Supplementary material for: PSAMM: A Portable System for the Analysis of Metabolic Models
Source: PLoS Comput Biol. 2016 Feb 1;12(2):e1004732. doi: 10.1371/journal.pcbi.1004732 (PMC4734835; doi:10.1371/journal.pcbi.1004732)
Supplement: S4 Text — The original version cannot provide a non-zero flux in FBA simulations, while the fixed version can. The problem was determined to be that there was no sink for the artificial biomass compound, M_BIOMASS, meaning it could accumulate but could not be counted towards biomass production. A sink was added for the compound allowing the model to generate a non-zero biomass flux. The lines are color coded to highlight the changes between the two versions. Highlighted in red are the lines removed, and highlighted in green are the lines added from the original version to the new version. (PDF) [file pcbi.1004732.s010.pdf]

S4 Text: Comparison of the original version and the fixed version of the model iMA871 [67] model using the *git diff* function in the Git version control system. The original version cannot provide a non-zero flux in FBA simulations, while the fixed version can. The problem was determined to be that there was no sink for the artificial biomass compound, *M_BIOMASS,* meaning it could accumulate but could not be counted towards biomass production. A sink was added for the compound allowing the model to generate a non-zero biomass flux. The lines are color coded to highlight the changes between the two versions. Highlighted in red are the lines removed, and highlighted in green are the lines added from the original version to the new version.

```
diff --git a/sbml/iMA871/medium.yaml b/sbml/iMA871/medium.yaml
index 57c3b57..c859836 100644
--- a/sbml/iMA871/medium.yaml
+++ b/sbml/iMA871/medium.yaml
@@ -419,3 +419,6 @@ compounds:
    reaction: R_1379
  - id: M_PROPALe
    reaction: R_1380
+- id: M_BIOMASS
+   compartment: C_c
+   lower: 0
```
