# Supplementary material for: PSAMM: A Portable System for the Analysis of Metabolic Models
Source: PLoS Comput Biol. 2016 Feb 1;12(2):e1004732. doi: 10.1371/journal.pcbi.1004732 (PMC4734835; doi:10.1371/journal.pcbi.1004732)
Supplement: S5 Text — The original version cannot provide a non-zero flux in FBA simulations. The new version of the model contained several changes including reaction directionality changes, stoichiometric changes, and the addition and deletion of reactions. The lines are color coded to highlight the changes between the two versions. Highlighted in red are the lines removed, and highlighted in green are the lines added from the original version to the new version. (PDF) [file pcbi.1004732.s011.pdf]

S5 Text: Comparison of the original version and the updated version (downloaded on September 09, 2015 from http://www.maranasgroup.com/models.htm) of the model iSyn731 [65] using the *git diff* function in Git version control system. The original version cannot provide a non-zero flux in FBA simulations. The new version of the model contained numerous changes including reaction directionality changes, stoichiometric changes, and the addition and deletion of reactions. The lines are color coded to highlight the changes between the two versions. Highlighted in red are the lines removed, and highlighted in green are the lines added from the original version to the new version.

```
diff --git a/sbml/iSyn731/reactions.yaml b/sbml/iSyn731/reactions.yaml
index 9434aa5..e77070e 100644
--- a/sbml/iSyn731/reactions.yaml
+++ b/sbml/iSyn731/reactions.yaml
@@ -39,7 +39,7 @@
     => |cpd00001_c[c]| + |cpd00006_c[c]| + |cpd03637_c[c]|'
 - id: BIOMASStex
   name: Biomass transport ext
-  equation: '|cpd11416_p[p]| <=> |cpd11416_e[e]|'
+  equation: '|cpd11416_p[p]| => |cpd11416_e[e]|'
 - id: Biomass_Auto
   name: Biomassformation under autotrophic condition
   equation: (53.4862) |cpd00002_c[c]| + (0.000044667) |cpd00004_c[c]| +
(0.0017867)
@@ -177,7 +177,7 @@
     + (38.3862) |cpd00009_c[c]|
 - id: Biomasstpp
   name: Biomass transport pp
-  equation: '|cpd11416_p[p]| <=> |cpd11416_c[c]|'
+  equation: '|cpd11416_c[c]| => |cpd11416_p[p]|'
 - id: CA
   name: Carbonic anhydrase
   equation: '|cpd00242_ca[ca]| + |cpd00067_ca[ca]| => |cpd00001_ca[ca]| +
|cpd00011_ca[ca]|'
@@ -245,9 +245,6 @@
 - id: CYTBDu
   name: 'cytochrome oxidase bd (plastocianine-8: 2 protons) (lumen)'
   equation: '|cpd16503_l[l]| + (0.5) |cpd00007_l[l]| => |cpd07274_l[l]| +
|cpd00001_l[l]|'
-- id: Copp
-  name: Carbon monoxide transport via diffusion (periplasm to cytosol)
-  equation: '|cpd00204_p[p]| => |cpd00204_c[c]|'
 - id: Cotpp
   name: Cobalt transport pp
   equation: '|cpd00149_p[p]| <=> |cpd00149_c[c]|'
@@ -359,16 +356,16 @@
   equation: '|cpd08376_e[e]| <=> |cpd08376_p[p]|'
 - id: GLCP
   name: glycogen phosphorylase
-  equation: '|cpd00009_c[c]| + |cpd00155_c[c]| => |cpd00089_c[c]|'
+  equation: '|cpd00009_c[c]| + (5) |cpd00155_c[c]| => |cpd00089_c[c]| +
|cpd00001_c[c]|'
 - id: GLCP2
   name: glycogen phosphorylase
-  equation: '|cpd00009_c[c]| + |cpd15413_c[c]| => |cpd00089_c[c]|'
+  equation: '|cpd00009_c[c]| + (5) |cpd15413_c[c]| => |cpd00089_c[c]| +
|cpd00001_c[c]|'
 - id: GLCtex
```

```
   name: Glucose transport ext
   equation: '|cpd00027_e[e]| <=> |cpd00027_p[p]|'
 - id: GLUttrappp
   name: TRAP system glutamate simport (periplasm)
-  equation: '|cpd00067_p[p]| + |cpd00023_p[p]| => |cpd00067_c[c]| +
|cpd00023_c[c]|'
+  equation: '|cpd00067_p[p]| + |cpd00023_p[p]| <=> |cpd00067_c[c]| +
|cpd00023_c[c]|'
 - id: GLYabcpp
   name: glycine transport via ABC system
   equation: '|cpd00002_c[c]| + |cpd00001_c[c]| + |cpd00033_p[p]| =>
|cpd00008_c[c]|
@@ -397,6 +394,9 @@
   name: Galactosyltransferase (n-C18:3)
   equation: '|cpd00043_c[c]| + |cp_DaLGl_c[c]| => |cpd00067_c[c]| +
|cpdMG183_c[c]|
     + |cpd00014_c[c]|'
+- id: Glycogentr
+  name: Glycogen transport ext
+  equation: '|cpd00155_e[e]| <=> |cpd00155_c[c]|'
 - id: H2ASE_syn
   name: Bidirectional [NiFe] Hydrogenase
   equation: '|cpd00067_c[c]| + |cpd00005_c[c]| <=> |cpd00006_c[c]| +
|cpd11640_c[c]|'
@@ -443,8 +443,8 @@
   equation: '|cpd00067_p[p]| + |cpd00130_p[p]| <=> |cpd00067_c[c]| +
|cpd00130_c[c]|'
 - id: MECDPDH_syn
   name: 2C-methyl-D-erythritol 2,4 cyclodiphosphate dehydratase
-  equation: '|cpd00005_c[c]| + |cpd08301_c[c]| => |cpd00001_c[c]| +
|cpd00006_c[c]|
-    + |cpd08615_c[c]|'
+  equation: '|cpd00005_c[c]| + |cpfdxr22_c[c]| + |cpd08301_c[c]| =>
|cpd00001_c[c]|
+    + |cpd00006_c[c]| + |cpfdxo22_c[c]| + |cpd08615_c[c]|'
 - id: MEHLER
   name: Mehler reaction
   equation: '|cpd00067_c[c]| + (0.5) |cpd00007_c[c]| + |cpd00005_c[c]| =>
|cpd00001_c[c]|
@@ -663,17 +663,13 @@
 - id: SDPSYN
   name: all-trans-nonaprenyl-diphosphate synthase [geranyl-diphosphate
specific]
   equation: '|cpd00283_c[c]| + (7) |cpd00113_c[c]| => |cpd02172_c[c]| + (7)
|cpd00012_c[c]|'
-- id: SERabcpp
-  name: L-serine transport via ABC system (periplasm)
-  equation: '|cpd00002_c[c]| + |cpd00001_c[c]| + |cpd00054_p[p]| =>
|cpd00008_c[c]|
-    + |cpd00067_c[c]| + |cpd00009_c[c]| + |cpd00054_c[c]|'
 - id: SPMDt2pp
   name: spermidine transport in via proton symport
   equation: '|cpd00067_p[p]| + |cpd00264_p[p]| => |cpd00067_c[c]| +
|cpd00264_c[c]|'
 - id: SQD2_181
   name: sulfoquinovosyltransferase  (n-C18:1)
   equation: '|cpd00001_c[c]| + |c_12dgr181_9_c[c]| + |cpd08358_c[c]| =>
```

```
|cpd00067_c[c]|
-     + |cpd00014_c[c]| + |sqdg181_c[c]|'
+     + |cpd00014_c[c]| + |cSQDG181_c[c]|'
 - id: SQD2_183
   name: sulfoquinovosyltransferase  (n-C18:3)
   equation: '|cpd00001_c[c]| + |c_12dgr183_6_9_12_c[c]| + |cpd08358_c[c]| =>
|cpd00067_c[c]|
@@ -697,7 +693,7 @@
 - id: SQVT_181
   name: Sulfoquinovosyltransferase (n-C18:1)
   equation: '|cpd00001_c[c]| + |cpd15312_c[c]| + |cpd08358_c[c]| =>
|cpd00067_c[c]|
-     + |cpd00014_c[c]| + |cSQDG181_c[c]|'
+     + |cpd00014_c[c]| + |sqdg181_c[c]|'
 - id: SQVT_182
   name: Sulfoquinovosyltransferase (n-C18:2)
   equation: '|cpd00001_c[c]| + |cp_DLiGl_c[c]| + |cpd08358_c[c]| =>
|cpd00067_c[c]|
@@ -709,9 +705,6 @@
 - id: SUCCtpp
   name: Succinate transport pp
   equation: '|cpd00067_p[p]| + |cpd00036_p[p]| <=> |cpd00067_c[c]| +
|cpd00036_c[c]|'
-- id: SUCD_tca
-  name: succinate dehydrogenase (TCA)
-  equation: '|cpd07274_p[p]| + |cpd00036_c[c]| => |cpd00106_c[c]| +
|cpd16503_p[p]|'
 - id: SUCDpp_syn
   name: succinate dehydrogenase (periplasm)
   equation: '|cpd07274_p[p]| + |cpd00036_c[c]| => |cpd00106_c[c]| +
|cpd16503_p[p]|'
@@ -733,10 +726,14 @@
   name: Urea transport via ABC system (periplasm)
   equation: '|cpd00002_c[c]| + |cpd00001_c[c]| + |cpd00073_p[p]| =>
|cpd00008_c[c]|
     + |cpd00067_c[c]| + |cpd00009_c[c]| + |cpd00073_c[c]|'
+- id: hyprxn
+  name: hyprxn
+  equation: '|cpd01855_c[c]| + |cpd00052_c[c]| + (2) |cpd00067_c[c]| =>
|cpd08287_c[c]|
+     + |cpd00012_c[c]| + |cpd00001_c[c]|'
 - id: molybtr
   name: Inducible molybdate transport system
-  equation: '|cpd00001_c[c]| + |cpd00002_c[c]| + |cpd11574_p[p]| =>
|cpd00008_c[c]|
-     + |cpd00009_c[c]| + |cpd11574_c[c]| + |cpd00067_c[c]|'
+  equation: '|cpd00001_c[c]| + |cpd00002_c[c]| + |cpd01174_p[p]| =>
|cpd00008_c[c]|
+     + |cpd00009_c[c]| + |cpd01174_c[c]| + |cpd00067_c[c]|'
 - id: rxn00001
   name: Pyrophosphate phosphohydrolase
   equation: '|cpd00001_c[c]| + |cpd00012_c[c]| => (2) |cpd00009_c[c]| + (2)
|cpd00067_c[c]|'
@@ -939,6 +936,9 @@
 - id: rxn00199
   name: Oxalosuccinate:NADP+ oxidoreductase (decarboxylating)
   equation: '|cpd00067_c[c]| + |cpd03187_c[c]| => |cpd00011_c[c]| +
```

```
|cpd00024_c[c]|'
+- id: rxn00203
+  name: 2-Oxoglutarate carboxy-lyase
+  equation: '|cpd00024_c[c]| + |cpd00067_c[c]| => |cpd00011_c[c]| +
|cpd00199_c[c]|'
 - id: rxn00205
   name: Glutathione:hydrogen-peroxide oxidoreductase
   equation: '|cpd00025_c[c]| + (2) |cpd00042_c[c]| <=> (2) |cpd00001_c[c]| +
|cpd00111_c[c]|'
@@ -1243,7 +1243,7 @@
   equation: '|cpd00002_c[c]| + |cpd00090_c[c]| <=> |cpd00008_c[c]| +
|cpd00068_c[c]|'
 - id: rxn00517
   name: ITP:pyruvate O2-phosphotransferase
-  equation: '|cpd00020_c[c]| + |cpd00068_c[c]| => |cpd00061_c[c]| +
|cpd00090_c[c]|'
+  equation: '|cpd00061_c[c]| + |cpd00090_c[c]| => |cpd00020_c[c]| +
|cpd00068_c[c]|'
 - id: rxn00525
   name: L-arogenate:NAD+ oxidoreductase
   equation: '|cpd00003_c[c]| + |cpd00616_c[c]| => |cpd00004_c[c]| +
|cpd00011_c[c]|
@@ -1435,7 +1435,7 @@
   equation: '|cpd00072_c[c]| + |cpd00102_c[c]| <=> |cpd00198_c[c]| +
|cpd00236_c[c]|'
 - id: rxn00786
   name: D-Fructose-1,6-bisphosphate D-glyceraldehyde-3-phosphate-lyase
-  equation: '|cpd00290_c[c]| <=> |cpd00095_c[c]| + |cpd00102_c[c]|'
+  equation: '|cpd00095_c[c]| + |cpd00102_c[c]| <=> |cpd00290_c[c]|'
 - id: rxn00789
   name: 1-(5-Phospho-D-ribosyl)-ATP:pyrophosphate phosphoribosyl-transferase
   equation: '|cpd00002_c[c]| + |cpd00103_c[c]| => |cpd00012_c[c]| +
|cpd01775_c[c]|'
@@ -1465,9 +1465,6 @@
 - id: rxn00826
   name: Glutathione:cystine oxidoreductase
   equation: (2) |cpd00042_c[c]| + |cpd00367_c[c]| => (2) |cpd00084_c[c]| +
|cpd00111_c[c]|
-- id: rxn00830
-  name: Isopentenyl-diphosphate delta3-delta2-isomerase
-  equation: '|cpd00113_c[c]| <=> |cpd00202_c[c]|'
 - id: rxn00832
   name: IMP 1,2-hydrolase (decyclizing)
   equation: '|cpd00001_c[c]| + |cpd00114_c[c]| <=> |cpd02884_c[c]|'
@@ -1561,12 +1558,12 @@
     + |cpd00129_c[c]|'
 - id: rxn00931
   name: L-Proline:NADP+ 5-oxidoreductase
-  equation: '|cpd00006_c[c]| + |cpd00129_c[c]| => |cpd00005_c[c]| +
|cpd00067_c[c]|
-    + |cpd02431_c[c]|'
+  equation: '|cpd00005_c[c]| + |cpd00067_c[c]| + |cpd02431_c[c]| =>
|cpd00006_c[c]|
+    + |cpd00129_c[c]|'
 - id: rxn00941
   name: Nicotinamide-nucleotide:pyrophosphate
-  equation: '|cpd00012_c[c]| + |cpd00355_c[c]| => |cpd00067_c[c]| +
```

```
|cpd00103_c[c]|
-    + |cpd00133_c[c]|'
+  equation: '|cpd00067_c[c]| + |cpd00103_c[c]| + |cpd00133_c[c]| <=>
|cpd00012_c[c]|
+    + |cpd00355_c[c]|'
 - id: rxn00947
   name: Palmitate:CoA ligase (AMP-forming)
   equation: '|cpd00002_c[c]| + |cpd00010_c[c]| + |cpd00067_c[c]| +
|cpd00214_c[c]|
@@ -1579,9 +1576,6 @@
   name: O-Acetyl-L-homoserine acetate-lyase (adding methanethiol)
   equation: '|cpd00239_c[c]| + |cpd00790_c[c]| => |cpd00067_c[c]| +
|cpd00029_c[c]|
     + |cpd00135_c[c]|'
-- id: rxn00966
-  name: chorismate pyruvate-lyase (4-hydroxybenzoate-forming)
-  equation: '|cpd00216_c[c]| => |cpd00020_c[c]| + |cpd00136_c[c]|'
 - id: rxn00967
   name: UDPglucose:4-hydroxybenzoate 4-O-beta-D-glucosyltransferase
   equation: '|cpd00026_c[c]| + |cpd00136_c[c]| <=> |cpd00014_c[c]| +
|cpd02476_c[c]|'
@@ -1706,8 +1700,8 @@
   equation: '|cpd00002_c[c]| + |cpd00190_c[c]| => |cpd00008_c[c]| +
|cpd00863_c[c]|'
 - id: rxn01184
   name: APS:ATP adenylyltransferase
-  equation: '|cpd00048_c[c]| + |cpd00067_c[c]| + |cpd00924_c[c]| =>
|cpd00002_c[c]|
-    + |cpd00193_c[c]|'
+  equation: '|cpd00002_c[c]| + |cpd00193_c[c]| => |cpd00048_c[c]| +
|cpd00067_c[c]|
+    + |cpd00924_c[c]|'
 - id: rxn01200
   name: Sedoheptulose-7-phosphate:D-glyceraldehyde-3-phosphate
   equation: '|cpd00102_c[c]| + |cpd00238_c[c]| <=> |cpd00101_c[c]| +
|cpd00198_c[c]|'
@@ -1799,7 +1793,7 @@
   equation: '|cpd00349_c[c]| <=> |cpd00095_c[c]| + |cpd00236_c[c]|'
 - id: rxn01343
   name: ATP:Sedoheptulose 7-phosphate 1-phosphotransferase
-  equation: '|cpd00002_c[c]| + |cpd00238_c[c]| <=> |cpd00008_c[c]| +
|cpd00349_c[c]|'
+  equation: '|cpd00002_c[c]| + |cpd00238_c[c]| => |cpd00008_c[c]| +
|cpd00349_c[c]|'
 - id: rxn01345
   name: Sedoheptulose 1,7-bisphosphate 1-phosphohydrolase
   equation: '|cpd00001_c[c]| + |cpd00349_c[c]| => |cpd00009_c[c]| +
|cpd00067_c[c]|
@@ -2127,9 +2121,6 @@
 - id: rxn02264
   name: Hydroxymethylbilane hydro-lyase(cyclizing)
   equation: '|cpd00755_c[c]| => |cpd00001_c[c]| + |cpd00774_c[c]|'
-- id: rxn02265
-  name: rxn02265
-  equation: '|cpd00755_c[c]| <=> |cpd00001_c[c]| + |cpd03414_c[c]|'
 - id: rxn02275
   name: 4-Guanidinobutanamide amidohydrolase
```

```
   equation: '|cpd00001_c[c]| + |cpd01973_c[c]| => |cpd00013_c[c]| +
|cpd00762_c[c]|'
@@ -2206,7 +2197,7 @@
   equation: '|cpd00863_c[c]| <=> |cpd00072_c[c]|'
 - id: rxn02402
   name: Nicotinate-nucleotide:pyrophosphate phosphoribosyltransferase
-  equation: '|cpd00011_c[c]| + |cpd00012_c[c]| + |cpd00873_c[c]| => (2)
|cpd00067_c[c]|
+  equation: '|cpd00011_c[c]| + |cpd00012_c[c]| + |cpd00873_c[c]| <=> (2)
|cpd00067_c[c]|
     + |cpd00103_c[c]| + |cpd02333_c[c]|'
 - id: rxn02465
   name: N-Acetyl-L-glutamate-5-semialdehyde:NADP+ 5-oxidoreductase
@@ -2653,6 +2644,10 @@
   name: L-phenylalanine,tetrahydrobiopterin:oxygen
   equation: '|cpd00007_c[c]| + |cpd00066_c[c]| + |cpd00233_c[c]| =>
|cpd00069_c[c]|
     + |cpd03897_c[c]|'
+- id: rxn04996
+  name: Glycogen transport ext
+  equation: '|cpd08615_c[c]| + |cpd00067_c[c]| + |cpd00005_c[c]| =>
|cpd00202_c[c]|
+    + |cpd00006_c[c]| + |cpd00001_c[c]|'
 - id: rxn05004
   name: S-adenosyl-L-methionine:gamma-tocopherol 5-O-methyltransferase
   equation: '|cpd00017_c[c]| + |cpd01631_c[c]| <=> |cpd00019_c[c]| +
|cpd00067_c[c]|
@@ -2718,10 +2713,6 @@
   name: TRDR
   equation: '|cpd00005_c[c]| + |cpd00067_c[c]| + |cpd11420_c[c]| =>
|cpd00006_c[c]|
     + |cpd11421_c[c]|'
-- id: rxn05293
-  name: MECDPDH
-  equation: '|cpd00004_c[c]| + |cpd00067_c[c]| + |cpd08301_c[c]| =>
|cpd00001_c[c]|
-    + |cpd00003_c[c]| + |cpd08615_c[c]|'
 - id: rxn05322
   name: Butyryl-[acyl-carrier protein]:malonyl-CoA
   equation: '|cpd00004_c[c]| + |cpd00067_c[c]| + |cpd11465_c[c]| =>
|cpd00003_c[c]|
@@ -3121,9 +3112,6 @@
 - id: rxn07980
   name: 3-hydroxyacyl-[acyl-carrier-protein] dehydratase (n-C18:1)
   equation: '|cpd15370_c[c]| => |cpd00001_c[c]| + |cpd15568_c[c]|'
-- id: rxn07981
-  name: 3-hydroxyacyl-[acyl-carrier-protein] dehydratase (n-C4:0)
-  equation: '|cpd11836_c[c]| => |cpd00001_c[c]| + |cpd11465_c[c]|'
 - id: rxn07987
   name: 3-oxoacyl-[acyl-carrier-protein] reductase (n-C12:1)
   equation: '|cpd00005_c[c]| + |cpd00067_c[c]| + |cpd15373_c[c]| =>
|cpd00006_c[c]|
@@ -3140,10 +3128,6 @@
   name: 3-oxoacyl-[acyl-carrier-protein] reductase (n-C18:1)
   equation: '|cpd00005_c[c]| + |cpd00067_c[c]| + |cpd15376_c[c]| =>
|cpd00006_c[c]|
     + |cpd15370_c[c]|'
```

```
-- id: rxn07994
-  name: 3-oxoacyl-[acyl-carrier-protein] reductase (n-C4:0)
-  equation: '|cpd00005_c[c]| + |cpd00067_c[c]| + |cpd11488_c[c]| <=>
|cpd00006_c[c]|
-    + |cpd11836_c[c]|'
 - id: rxn07997
   name: 3-oxoacyl-[acyl-carrier-protein] synthase (n-C12:1)
   equation: '|cpd00067_c[c]| + |cpd11492_c[c]| + |cpd15416_c[c]| =>
|cpd00011_c[c]|
@@ -3224,9 +3208,6 @@
 - id: rxn08155
   name: L-arginine transport via diffusion (extracellular to periplasm)
   equation: '|cpd00051_e[e]| <=> |cpd00051_p[p]|'
-- id: rxn08164
-  name: arsenite transport via diffusion (extracellular to periplasm)
-  equation: '|cpd04098_e[e]| <=> |cpd04098_p[p]|'
 - id: rxn08171
   name: arsenate reductase
   equation: (2) |cpd00042_c[c]| + (2) |cpd00067_c[c]| + |cpd01048_c[c]| <=>
|cpd00001_c[c]|
@@ -3785,9 +3766,6 @@
   name: pyridoxal oxidase
   equation: (2) |cpd00001_c[c]| + (0.5) |cpd00007_c[c]| + |cpd00013_c[c]| +
|cpd00215_c[c]|
     <=> (2) |cpd00025_c[c]| + |cpd00419_c[c]|
-- id: rxn09693
-  name: Biotin uptake
-  equation: '|cpd00067_e[e]| + |cpd00104_e[e]| <=> |cpd00067_c[c]| +
|cpd00104_c[c]|'
 - id: rxn09753
   name: fumarate reductase, cytosolic/mitochondrial
   equation: '|cpd00106_c[c]| + |cpd00982_c[c]| => |cpd00015_c[c]| +
|cpd00036_c[c]|'
@@ -3874,7 +3852,7 @@
     + (3) |cpd00067_c[c]| + (2) |cpfdxr22_c[c]|'
 - id: rxn10481
   name: Copper transport via ABC system
-  equation: '|cpd00001_c[c]| + |cpd00002_c[c]| + |cpd00058_p[p]| =>
|cpd00008_c[c]|
+  equation: '|cpd00001_c[c]| + |cpd00002_c[c]| + |cpd00058_e[e]| =>
|cpd00008_c[c]|
     + |cpd00009_c[c]| + |cpd00058_c[c]| + |cpd00067_c[c]|'
 - id: rxn10481_1
   name: Copper (Cu+2) ABC transporter (periplasm)
@@ -3947,9 +3925,6 @@
   name: pantothenate kinase
   equation: '|cpd00002_c[c]| + |cpd00644_c[c]| => |cpd00008_c[c]| +
|cpd00067_c[c]|
     + |cpd02201_c[c]|'
-- id: rxn12549
-  name: 4-Hydroxybenzoate transport via proton symport
-  equation: '|cpd00067_e[e]| + |cpd00136_e[e]| <=> |cpd00067_c[c]| +
|cpd00136_c[c]|'
 - id: rxn12770
   name: (3R)-3-Hydroxyacyl-CoA:NADP+ oxidoreductase
   equation: '|cpd00005_c[c]| + |cpd03123_c[c]| <=> |cpd00006_c[c]| +
|cpd16757_c[c]|'
```

```
@@ -3962,7 +3937,7 @@
     => (2) |cpd00023_c[c]| + (2) |cpfdxo22_c[c]|
 - id: rxn12825
   name: Heme A synthase
-  equation: '|cpd00001_c[c]| + |cpd11313_c[c]| => (4) |cpd00067_c[c]| +
|cpd00028_c[c]|'
+  equation: '|cpd00001_c[c]| + |cpd11313_c[c]| => (4) |cpd00067_c[c]| +
|cpd11312_c[c]|'
 - id: rxn13643
   name: glycogen synthase (ADPGlc)
   equation: '|cpd00387_c[c]| => |cpd00008_c[c]| + |cpd00067_c[c]| +
|cpd00155_c[c]|'
@@ -3970,9 +3945,6 @@
   name: 1,4-alpha-glucan branching enzyme
   equation: (4) |cpd00002_c[c]| + (5) |cpd00027_c[c]| => (4) |cpd00067_c[c]|
+ |cpd00008_c[c]|
     + (4) |cpd00009_c[c]| + (4) |cpd00001_c[c]| + |cpd00155_c[c]|
-- id: rxn13759
-  name: Arsenite transporter
-  equation: '|cpd00067_e[e]| + |cpd01048_e[e]| <=> |cpd00067_c[c]| +
|cpd01048_c[c]|'
 - id: rxn13840
   name: (3R)-3-Hydroxypalmitoyl-[acyl-carrier-protein] hydro-lyase
   equation: '|cpd11481_c[c]| <=> |cpd00001_c[c]| + |cpd15239_c[c]|'
@@ -3982,3 +3954,7 @@
 - id: rxn_3pgctr
   name: Diffusion transport
   equation: '|cpd00169_ca[ca]| => |cpd00169_c[c]|'
+- id: xylcycle
+  name: xylcycle
+  equation: '|cpd00198_c[c]| + |cpd00067_c[c]| + |cpd00005_c[c]| =>
|cpd01855_c[c]|
+    + |cpd00006_c[c]|'
```
